# Dissecting minimal residual disease dynamics to improve outcome prediction in mantle cell lymphoma: Data from the Fondazione Italiana Linfomi (FIL)‐MCL0208 clinical trial

**DOI:** 10.1002/hem3.70375

**Published:** 2026-04-30

**Authors:** Francesca Cordero, Simone Ferrero, Simone Pernice, Elisa Genuardi, Daniela Volpatto, Roberta Sirovich, Aurora Maria Civita, Andrea Evangelista, Simone Ragaini, Alice Di Rocco, Alessandro Re, Vittorio Stefoni, Federica Cavallo, Carola Boccomini, Monica Balzarotti, Vittorio Ruggero Zilioli, Maria Gomes da Silva, Luca Arcaini, Melania Celli, Gian Maria Zaccaria, Dora Tortarolo, Marco Beccuti, Eva Hoster, Christiane Pott, Elizabeth Macintyre, Olivier Hermine, Martin Dreyling, Marco Ladetto

**Affiliations:** ^1^ Department of Computer Science University of Turin Torino Italy; ^2^ Hematology Division, Department of Molecular Biotechnologies and Health Sciences University of Turin Torino Italy; ^3^ Hematology Unit Azienda Ospedaliero‐Universitaria “Città della Salute e della Scienza di Torino” Torino Italy; ^4^ Department of Mathematics “G. Peano” University of Turin Torino Italy; ^5^ Unit of Clinical Epidemiology Azienda Ospedaliera Universitaria Città della Salute e della Scienza and CPO Piemonte Torino Italy; ^6^ Department of Translational and Precision Medicine Sapienza University of Rome Rome Italy; ^7^ Department of Haematology ASST‐Spedali Civili Hospital Brescia Italy; ^8^ Institute of Haematology L. e A. Seràgnoli University of Bologna Bologna Italy; ^9^ Department of Molecular Biotechnologies and Health Sciences Universitaria Città della Salute e della Scienza di Torino and Centre for Cancer Prevention Piemonte Torino Italy; ^10^ SC Ematologia AOU “Città della Salute e della Scienza di Torino” Torino Italy; ^11^ Hematology Unit IRCCS Humanitas Research Hospital, Rozzano Milan Italy; ^12^ Division of Hematology ASST Grande Ospedale Metropolitano Niguarda Milan Italy; ^13^ Departamento de Hematologia Instituto Portugues de Oncologia de Lisboa Lisbon Portugal; ^14^ Department of Molecular Medicine University of Pavia Pavia Italy; ^15^ Division of Hematology Fondazione IRCCS Policlinico San Matteo Pavia Italy; ^16^ Ospedale Degli Infermi Di Rimini Rimini Italy; ^17^ Department of Electrical and Information Engineering (DEI) Polytechnic University of Bari Bari Italy; ^18^ Institute of Medical Informatics, Biometry, and Epidemiology Ludwig‐Maximilians‐University of Munich Munich Germany; ^19^ Second Medical Department University Hospital Schleswig‐Holstein Kiel Germany; ^20^ INSERM U1151, Institut Necker Enfants Malades (INEM) Paris France; ^21^ Laboratory of Onco‐Hematology Necker Children's Hospital, Assistance Publique‐Hôpitaux de Paris (AP‐HP) Paris France; ^22^ Université Paris Cité Paris France; ^23^ Service d'Hématologie Adulte Hôpital Universitaire Necker‐Enfants Malades, APHP Université Paris Cité Paris France; ^24^ Department of Hematology INSERM U1163, IMAGINE Institute, Hôpital Universitaire Necker Enfants‐Malades Université Paris Cité Paris France; ^25^ Department of Medicine III LMU Hospital Munich Germany; ^26^ Division of Hematology Antonio e Biagio e Cesare Arrigo Hospital Alessandria Italy

## Abstract

Recent clinical trials have underscored the value of repeated minimal residual disease (MRD) measurements as a highly sensitive method for detecting subclinical disease and enabling dynamic risk stratification in hematologic malignancies. Despite its clinical potential, the complex and heterogeneous nature of MRD kinetics presents significant challenges for interpreting and integrating it into routine clinical decision‐making. In this study, we present a comprehensive, model‐based workflow for the longitudinal analysis of MRD trajectories designed to improve relapse risk prediction. We applied this newly developed workflow to a cohort of patients with mantle cell lymphoma (MCL). MRD measurements were collected from both bone marrow (BM) and peripheral blood (PB) over time, stored in the Fondazione Italiana Linfomi MCL0208 clinical trial. Using our functional MRD workflow, we defined four MRD dynamics that collapsed into two clinically relevant groups: favorable (rapid, sustained negativization) and unfavorable (persistent or fluctuating MRD). Patients with unfavorable profiles showed significantly shorter time to progression (TTP), with hazard ratio (HR) = 4.18 (95% CI: 2.44–7.14) in BM and HR = 5.71 (95% CI: 2.86–11.42) in PB. External validation in the European MCL Network “Younger trial” confirmed the predictive power of this stratification, with Kaplan–Meier analyses demonstrating significant prognostic discrimination. The most informative temporal windows for patient clustering vary by tissue. Early‐phase BM assessments offer greater discriminatory power, whereas late‐phase assessments are most informative in PB. These findings indicate that longitudinal MRD assessment in PB represents a clinically actionable strategy that could reduce dependence on invasive BM procedures.

## Graphical abstract

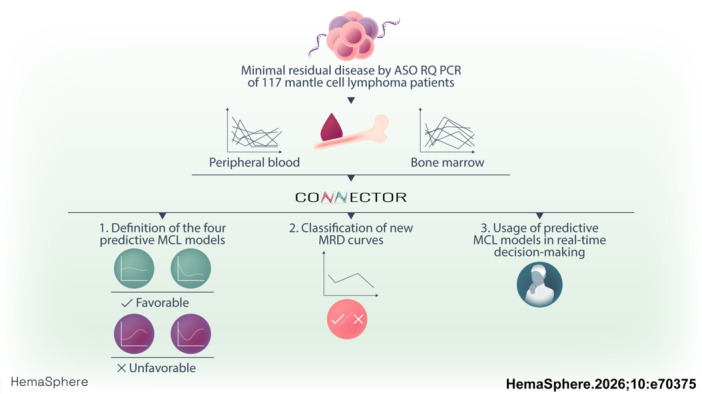

## INTRODUCTION

Minimal residual disease (MRD) refers to the small number of cancer cells that persist in a patient during or after treatment. It is typically identified through the analysis of biological samples collected at various time points. Above all, when there are no clinical or radiological signs of disease, assessing MRD requires highly sensitive methods capable of detecting these residual tumor cells. This is especially important for evaluating the effectiveness of both conventional and experimental therapies. MRD assessment provides valuable feedback on treatment response and serves as a strong prognostic indicator of clinical outcomes.^1^ It has been primarily studied in blood cancers, where its significance in disease monitoring has been well established.

Considerable effort has been devoted to developing practical tools for identifying patients at high risk of relapse.^2^ A pivotal example is the monitoring of MRD during and after treatment in mantle cell lymphoma (MCL).^3^ MCL is a rare and aggressive form of B‐cell non‐Hodgkin lymphoma, accounting for approximately 6% of non‐Hodgkin lymphoma cases. While MCL is generally characterized by initial clinical responses following induction therapy, an early relapse pattern is frequently observed. Despite significant advances in treatment outcomes, MCL remains an incurable disease, with a continuous pattern of subsequent disease relapse and a median survival of approximately 6–9 years.^4^

Among recognized prognosticators in MCL, MRD has gained considerable interest following the publication of several reports describing its high predictive value in this lymphoma subtype.^5^, ^6^, ^7^ In particular, a prospective clinical trial recently described the clinical relevance of repeated MRD monitoring.^6^, ^8^, ^9^

Latest technical developments in MRD detection have supported its establishment as a widely feasible and standardized tool for the direct assessment of therapy‐induced tumor burden reduction.^10^ Among the main findings reported, allele‐specific oligonucleotide real‐time quantitative polymerase chain reaction (ASO RQ‐PCR) is the most reliable and standardized technique^11^ for performing robust MRD detection and prognostication in MCL. Nonetheless, complex patterns of MRD kinetics over time and the availability of results obtained by heterogeneous tissues generate substantial interpretation issues and hamper an easy‐to‐use application of this predictive biomarker. According to Ferrero et al.,^6^ relying on a single MRD time point is not sufficient to predict the future course of such a chronic, remitting disease. Accordingly, a time‐varying kinetic model was implemented to interpret MRD better, enhancing the capacity to predict outcomes in MCL patients. This approach is based on an extension of the Cox model designed to incorporate time‐dependent effects. Indeed, longitudinal “kinetic” monitoring (where stable MRD positivity or fluctuating positive/negative MRD results are observed) is a stronger predictor of disease progression than the fixed “snapshot” offered from a single MRD time point. On the other hand, consistently negative MRD results are associated with a lower risk of relapse. Traditionally, MRD studies have focused on describing the temporal evolution of the disease through the phenomenological categorization of MRD markers monitored during and after treatment. This conventional approach typically simplifies the analysis into a dichotomous view, classifying MRD results as either positive or negative at specific time points. In the context of the FIL‐MCL0208 trial (clinicaltrials.gov no. 02354313), a Phase III study evaluating the efficacy of lenalidomide maintenance versus observation,^1^ recent efforts have shifted toward the consideration of the entire series of MRD positive/negative values collected over time, rather than focusing on isolated time points.^5^, ^12^ The value of MRD assessment in improving outcome prediction beyond conventional prognostic factors has been demonstrated in several studies, as has the critical role of MRD detection in MCL for guiding personalized treatment strategies.^13^, ^14^, ^15^

Taking advantage of the comprehensive dataset of systematic MRD assessment offered by the FIL‐MCL0208 trial,^1^ we further improved and automatized new predictive models, considering the whole MRD history of each patient, independently from the acquisition of the other clinical and outcome data. In contrast to the limited dichotomous view, we proposed the functional MRD workflow, a model‐based approach to MRD analysis that enables a more comprehensive investigation of the dynamic behavior of the systems over time by considering the entire set of time points, even when these are collected irregularly and sparsely. Indeed, our approach is able to recover continuous trajectories from discrete time‐point measurements, effectively capturing the underlying mechanisms driving temporal changes.

First, we defined tissue‐based MCL predictive models training the model on FIL0208. The predictive models were generated by the application of CONNECTOR,^16^ a novel automated computational framework, to refine the interpretation of MRD kinetics and to stratify patients into novel risk classes based on a solid, algorithm‐derived classification. CONNECTOR conducts an unsupervised longitudinal data analysis, excelling in cases where observations are sparse and irregularly spaced. The mixed‐effects model utilized in CONNECTOR can accommodate non‐linear relationships and interactions, providing significant flexibility to analyze the complex temporal dynamics of MRD.

To further enhance the predictive power of these MCL predictive models, we utilized an independent validation cohort,^8^ showcasing their ability to predict outcomes even outside our original patient series and thus their potential scalability.

In addition, we also incorporated the available clinical and biological features already associated with the MCL prognostication to explore their stratification capabilities. This includes evaluating longitudinal time points, considering the time points analyzed, and integrating data from tissues such as bone marrow (BM) and peripheral blood (PB). All results can now be easily explored using the *user‐friendly* MCL Explorer Shiny app. This application allows users to browse the results and provides the functionality to classify their own MRD data, enabling them to automatically associate their real patients with one of the described predictive clusters.

This study led to the generation of a predictive and practical, easy to use, framework for stratifying MCL patients into groups on the basis of subsequently acquired MRD determinations. Our framework is also able to highlight the importance of specific time points for MRD assessment in MCL, leading to the definition of optimal MRD timing. This work represents a crucial step toward developing software for real‐time applications, such as predicting patient outcomes. The validation proposed in this study should serve as a driving force for integrating MRD analysis into clinical practice for predicting disease progression.

## MATERIALS AND METHODS

### The workflow phases

The functional MRD workflow presented here is designed to identify and exploit predictive models for quantitative measurements of MRD collected over time. We rely on the literature in functional data analysis, particularly for functional data clustering and classification. In this context, we interpret data as observations with a longitudinal structure, sampled at discrete time points from a heterogeneous population of curves (organized into subpopulations or groups). Thus, the MRD quantitative measurements obtained from a single subject can be regarded as observations of an underlying continuous trajectory that is only observed at discrete time points. Measurements collected across multiple subjects constitute the overall dataset. The proposed workflow identifies a statistical model describing the temporal evolution of MRD that accounts for two sources of variability: individual‐level and group‐level (or cluster‐level), where subjects are assigned to groups based on a similarity criterion.

**Figure 1:**
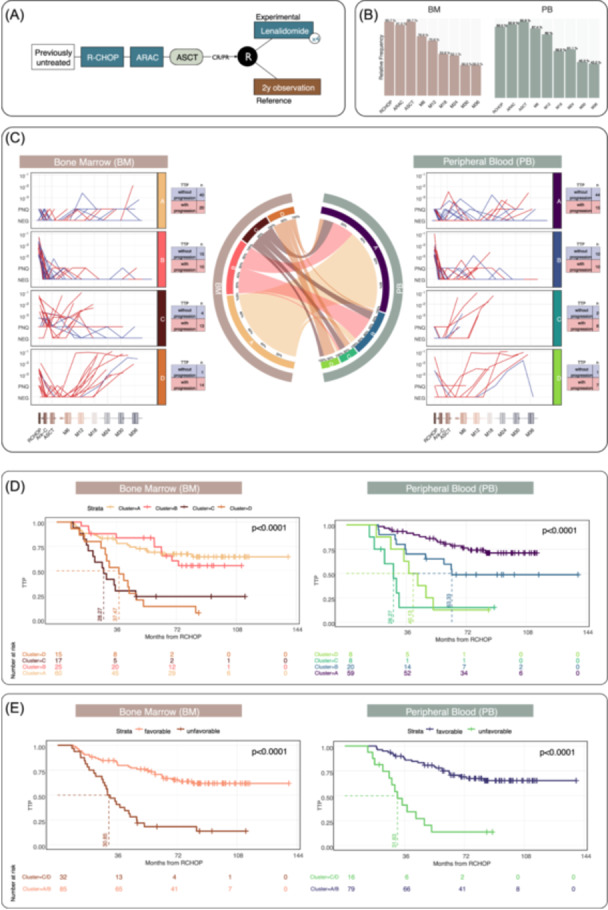
**Definition of mantle cell lymphoma (MCL) predictive models. (A)** The schema of the FIL‐MCL0208 trial. **(B)** Frequency distribution of the collected samples at each time point along the treatment timeline. **(C)** The CONNECTOR bone marrow (BM) and peripheral blood (PB) minimal residual disease (MRD) data analysis obtained four clusters. The chord diagram describes the transition from BM‐associated clusters to PB‐associated clusters. **(D)** The Kaplan–Meier survival curves (time from RCHOP) stratifying the MCL patients by the CONNECTOR clusters. The dashed lines and the corresponding numbers indicate the median survival for each group, defined as the time at which the estimated survival probability falls below 0.5. **(E)** Kaplan–Meier curves of the two groups of patients with favorable (Cluster A and Cluster B) versus unfavorable (Cluster C and Cluster D) dynamics.

The workflow consists of four phases: *pre‐processing data*, *predictive model definition*, *model abstraction* (a new function of the tool CONNECTOR), and *post‐processing statistical analysis*. Supporting Information S1: Figure 1 summarizes the workflow.

The *pre‐processing* phase is dedicated to importing, exploring, cleaning, transforming, visualizing, and filtering data. It includes all the steps that lead to well‐structured and ready‐for‐more sophisticated analyses or modeling data.

The *predictive model definition* phase has the core objective of fitting a statistical model while accounting for clustering the sampled population into groups. Indeed, the longitudinal data are analyzed through a model‐based clustering algorithm for functional data. The model includes the cluster memberships, which are treated as missing and estimated. The task is accomplished using the CONNECTOR tool, see Ref. 16 for a complete overview of the framework. The output includes reconstructed MRD curves at any time point and estimated cluster membership probabilities for each subject. Therefore, once the CONNECTOR tool estimates the statistical model of MRD evolution, the outcome is twofold. First, it provides an estimate of the MRD trajectory at any time point, derived from information across the entire cohort. Second, each set of MRD measurements collected at a limited number of time points from an individual subject is assigned to the cluster with the largest membership probability. Consequently, each subject is stratified into a specific group according to their MRD evolution pattern. We show here that the varying dynamics of the reconstructed MRD curves across the identified clusters provide insights into potential disease progression in different patient groups.

The *model abstraction* phase uses the predictive model from the previous step to classify never‐before‐seen MRD measurements, predicting their group membership and full dynamics. Indeed, once the statistical model has been estimated, it can be leveraged to generate predictions for both the MRD trajectory at any time point and the probabilities of cluster membership. At the end of this phase, the new MRD measurements from the new subjects are enriched with full trajectories and cluster assignments. Details of the method are provided in Supporting Information S1: Section 1.1. This process was integrated into the CONNECTOR tool, creating a new *classification module component*, which represents a novel contribution of this work.

After clustering and classification, the *post‐processing statistical analysis* phase examines the relationships between the predicted clusters and other available subject‐related information. The statistical analysis includes survival and landmark analyses to assess the prognostic relevance of the new subject stratification for the time‐to‐progression (TTP) outcome. Moreover, the MCL evolution models were thoroughly assessed by integrating all available clinical data, including clinical parameters, mutational profiles, and adverse treatment effects. A comprehensive description of each phase of the workflow is provided in Supporting Information S1: Sections 1.1 and 1.2.

### MCL Explorer: A Shiny application

We developed MCL Explorer, an interactive Shiny application designed for analyzing, inspecting, and visualizing the clustering and classification results presented in the paper. The application is available at https://github.com/qBioTurin/MCLexplorer and is built with the Shiny package in R, providing a structured, user‐friendly interface. The MCL Explorer dashboard includes a navigation menu with three main sections. The first, Clustering Exploration, enables users to examine clustering results through entropy analysis. The second, Classification Exploration, focuses on exploring classification outcomes derived from the MCL Younger trial dataset. The third, Classify Your Data, allows users to upload their own datasets and perform classifications. This last section is divided into two parts: Upload, where users can upload and preview their data, and Classification, where they can run the analysis.

### FIL‐MCL0208 cohort

The MCL predictive models were generated from the FIL‐MCL0208 trial (ClinicalTrials.gov identifier: NCT02354313), a multicenter, open‐label, randomized Phase III study evaluating the efficacy and safety of lenalidomide maintenance (experimental arm) versus observation (reference arm) following autologous stem cell transplantation (ASCT). The trial enrolled 300 patients across Italy and Portugal, and the clinical results have already been published.^17^ The treatment consisted of an induction phase (three cycles of RCHOP, given every 21 days), a consolidation phase composed of high‐dose cyclophosphamide and rituximab (R‐CTX), and two cycles of high‐dose Ara‐C and rituximab (R‐HD‐ARAC), BEAM conditioning regimen, and ASCT. In the experimental arm (LEN), lenalidomide was administered at 15 mg once daily on Days 1–21 every 28 days for 2 years, while in the reference arm (OBS), no therapy was administered.

The FIL‐MCL0208 trial included systematic monitoring of MRD in both PB and BM using nested PCR and ASO RQ‐PCR at 10 defined time points to detect immunoglobulin heavy chain (*IGH*) clonal rearrangements and the *BCL1/IGH* major translocation cluster, as previously described.^6^ BM and PB were collected at diagnosis and follow‐up according to the following clinical restaging time points: (1) the induction phase after R‐CTX; (2) the consolidation phase after R‐HD‐ARAC; (3) post‐ASCT; (4) during LEN or OBS, every 6 months (M6‐12‐18‐24); and (5) during FU (M30‐36). A high level of sampling compliance was recorded in the MCL0208 trial. Specifically, 90%–95% of BM and PB samples MRD time points were collected within post‐ASCT, and 65%–70% were collected during maintenance and follow‐up. Details of the overall dataset are reported in Supporting Information S1: Section 1.3.

## RESULTS

### Definition of the predictive MCL models

The MRD data that underwent the first phase of the functional MCL workflow are obtained from the FIL‐MCL0208 trial illustrated in Figure 1A. From the original 300 patients, a total of 117 MRD profiles with at least three recorded time points in BM tissue (median: 6; range: 3–9) and 95 MRD profiles with at least four recorded time points in PB tissue (median: 7; range: 4–9) were included in the analysis. Notably, paired MRD samples from BM and PB were available for 95 patients and analyzed. The baseline measurement is not considered an MRD time point; therefore, the minimum number of recorded time points begins after the R‐CHOP treatment phase. Figure 1B illustrates the distribution of collected time points along the treatment timeline. Notably, an average of 94% (93%) of patients' BM (PB) samples have been recorded by the first three time points (R‐CHOP, ARAC, and ASCT). The full details about the import, cleaning, and transformation of the data are reported in Supporting Information S1: Section 2.2.

The model definition phase is based on MRD analysis using the CONNECTOR framework, which enables the unsupervised stratification of patients into distinct groups without relying on clinical assumptions or outcome data. Longitudinal MRD measurements from both BM and PB samples are analyzed separately.

The functional clustering model is dependent on the selection of free parameters. To support users in this process, CONNECTOR includes an integrated toolset that guides the configuration of these parameters appropriately. Based on the optimal values suggested by the implemented evaluation indices, four clusters are identified in both the BM and PB datasets (Figure 1C). A comprehensive CONNECTOR report detailing all selected parameters is provided in Supporting Information S1: Sections 1.3 and 2.2 and illustrated in Supporting Information S1: Figures 3 and 4.

Figure 1C (left column) illustrates the clustering of the MRD longitudinal measurements from BM MRD data. Cluster BM_A, *stable‐negative*, comprises 60 patients whose MRD showed rapid and stable negativization, with initial measurements (R‐CHOP) either at a non‐quantifiable positive level (i.e., 10^−6^) or with low‐level MRD persistence. Cluster BM_B comprises 25 patients with high MRD levels during the initial treatment phases (R‐CHOP/Ara‐C steps), followed by MRD negativization at ASCT. In BM_A and BM_B, the majority of patients did not experience a relapse (40 in BM_A and 15 in BM_B), while a smaller subset did relapse (20 in BM_A and 10 in BM_B). Cluster BM_C includes 17 patients who primarily exhibited a wide range of MRD values during the initial phase, followed by either fluctuations in MRD levels or reappearance of MRD after an early period of negativization. In contrast, three trajectories within this cluster are characterized by a stable trend or oscillations between PNQ (positive non‐quantifiable) and NEG (negative) values. Cluster BM_D includes 15 patients with high initial MRD levels, followed by only transient MRD negativization and later MRD reappearance. In these two clusters, the vast majority of patients experienced relapse (13 in BM_C and 14 in BM_D), compared to the few who did not (4 in BM_C and 1 in BM_D). The Kaplan–Meier plot revealed that the median TTP has not yet been reached for BM_A and BM_B. In contrast, the median TTP is 36 months for BM_C and 27 months for BM_D, see Figure 1D. The estimated survival functions for the four CONNECTOR clusters differ significantly (P < 0.0001).

The model estimated independently by CONNECTOR on PB samples generated a similar stratification of the patients, Figure 1C (right column). Specifically, the first two clusters, PB_A and PB_B, showed a stable MRD negativization or an MRD alternating trend. In contrast, PB_C and PB_D clusters contained patients characterized by early and late MRD reappearance. Figure 1D depicts the Kaplan–Meier curves stratified by PB clusters. The median TTP is not yet reached for PB_A, while in PB_B it is reached at 60 months, in PB_C at 41 months, and in PB_D at 28 months. The survival functions estimated for the four CONNECTOR clusters show significant differences (P < 0.0001).

Observing the distribution of relapsed patients and the MRD impact stratifying the patients into the four clusters suggests the definition of two behaviors: the favorable MRD kinetics include joined Clusters A and B from both tissues, with a median TTP not reached. The unfavorable MRD kinetics include merged Clusters C and D from BM and PB tissues, respectively, with a median TTP of 30 and 31 months (P < 0.0001), Figure 1D. Thus, the converter identifies two different prognostic groups: Favorable MRD—characterized by rapid and stable MRD negativization, and, considering both tissues, the median TTP value exceeds the study. Unfavorable MRD—marked by fluctuating MRD level characterized by the median value of TTP to 2.57 years (BM) and 2.64 years (PB), with log‐rank P < 0.0001 concerning the favorable MRD. The Kaplan–Meier curves are illustrated in Figure 1E. The TTP hazard ratio (HR) of patients in the unfavorable MRD cluster is 4.18 (CI 95% 2.44–7.14) in BM and HR = 5.71 (CI 95% 2.86–11.42) in PB.

The chord diagram in Figure 1C illustrates the flow of 95 patients for whom MRD was measured in both BM and PB, showing the transition from BM‐ to PB‐associated clusters. To support the identification of two MCL MRD kinetic patterns, the diagram highlights that most patients in BM_A and BM_B are associated with cluster PB_A (approximately 80% and 40%, respectively) or PB_B (20% and 55%, respectively). Conversely, patients in clusters BM_C and BM_D exhibit a less consistent association with PB_C (17% and 20%, respectively) and PB_D (11% and 40%, respectively). Overall, patient allocation to BM and PB clusters was concordant for 89% of subjects, indicating strong consistency between the two tissue‐based clustering patterns. To adopt a conservative clinical approach, we performed the analysis presented in this section. In addition, we also analyzed MRD trajectories comprising only two time points; the corresponding results are reported in Supporting Information S1: Section 2.8.

### Post‐processing and overall clinical assessment of MCL predictive models

In this section, we discuss qualitative characteristics of the resulting MCL predictive models, and we link the cluster associations to other surveyed clinical variables. All results collected are easily browsed in the MCL Explorer (https://github.com/qBioTurin/MCLexplorer), see Supporting Information S1: Section 1.2.

#### Uncertainty of MRD dynamics

The MCL predictive models estimate the cluster membership distribution and assign each subject to the class with the largest probability. To measure the uncertainty in the allocation to a specific cluster, we explore the Shannon entropy of the cluster membership distribution.

**Figure 2:**
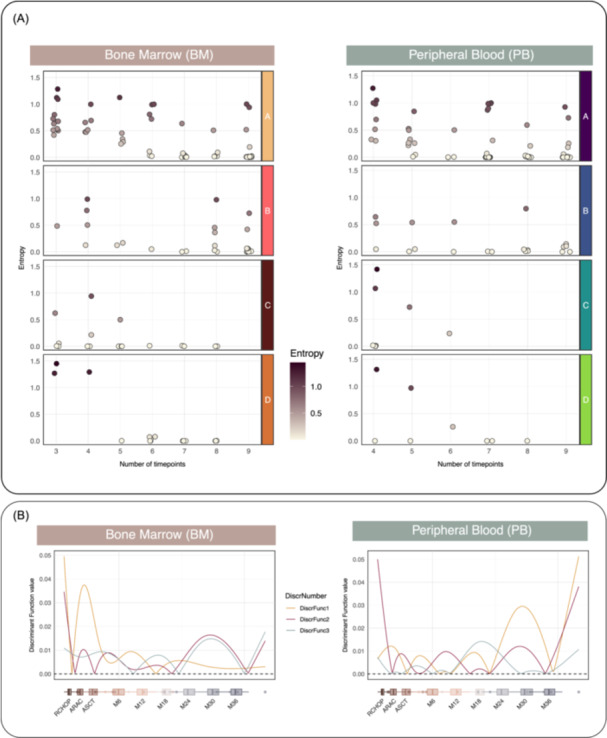
**(A) In both columns, the four scatter plots show the entropy values associated with each minimal residual disease (MRD) curve (vertical axis) distributed over the number of time points of the longitudinal measurement (horizontal axis).** Larger values of entropy correspond to increased uncertainty of the cluster assignment. **(B)** Line plots represent the discriminant function. Higher discriminant function values reflect a greater influence of the corresponding time points on cluster assignment.

Figure 2A displays eight scatter plots (one for each BM/PB cluster), showing that higher entropy values, which are associated with higher uncertainty in the cluster membership selection, are generally associated with MRD longitudinal measurements composed of less than six time points.

#### MRD clusters and response to treatment

Observing the BM MRD longitudinal measurements clustered in the four CONNECTOR groups, it is clear that in BM_A, there are all measurements characterized by a value at RCHOP negative or positive not quantifiable (PNQ), while in BM_B, there are all measurements whose RCHOP is equal to an MRD positivity. Otherwise, the main difference between BM_C and BM_D is appreciable, considering the length of the negativization period explaining the discrimination role of the M24 and M30 time points. The same considerations are extendable to the PB clusters. The model‐based approach used in the *predictive model definition* phase of the workflow enables exploration of the discriminant functions to assess the importance of each time point in the model estimation, see Refs. 16 and 18 for the technical details. Considering that higher function values indicate greater importance of the corresponding time intervals to the cluster assignment, the BM discriminant functions plotted in Figure 2B (left column) suggest that the RCHOP time point is strongly discriminative and that the interval between ARAC and ASCT is crucial for defining the MCL predictive model. Moreover, the plot suggests that the time interval between M24 and M30 also shows high discriminative power during the maintenance phase. Moving to the PB tissue, the PB discriminant functions plotted in Figure 2B (right column) show concordance on the importance of the RCHOP time point and strengthen the relevance of the time interval between M24 and M30.

#### Clinical characteristics of identified MRD clusters

All collected variables are considered when assessing the MCL predictive models. Specifically, the clinical relevance of the identified BM and PB clusters was assessed using 27 clinical and biological variables (listed in Supporting Information S1: Tables 2 and 3), as well as 11 specific germline polymorphisms of transmembrane transporters, metabolic enzymes, and cell‐surface receptors (ABCB1, ABCG2, VEGFA, FCGR2A, NCF4, GSTP1, and CRBN).

No significant differences were found. Additionally, we tested the cluster against a panel of five genes (BIRC3, NOTCH1, ATM, KMT2D, and TP53) using FFPE samples derived from lymph nodes; variants were called with Mutect2, annotated using Franklin Genox, and a gene was considered “mutated” if it carried any pathogenic or likely pathogenic variant. Again, no gene mutation correlated with the cluster association. The effect of lenalidomide maintenance was also examined to further dissect the results. In Supporting Information S1: Figure 7, BM clusters are additionally stratified based on maintenance treatment received, grouping patients into the reference (observation) or the experimental (lenalidomide) arm. The Kaplan–Meier plot, which stratifies patients within each cluster by treatment arm, highlights differences in TTP in clusters BM_A and BM_C. Specifically, in cluster BM_C, nearly all patients in the observational arm experienced relapse except for one. In contrast, in the experimental arm, only two out of five patients relapsed.

**Figure 3:**
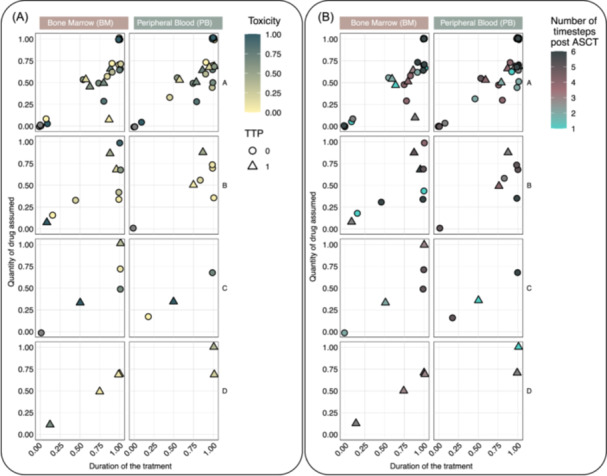
**The relationship between treatment adherence and disease progression across mantle cell lymphoma (MCL) clusters is explored, considering early discontinuation or dose reduction of Lenalidomide due to hematologic toxicity.** Treatment quantification and duration were standardized, and all details are provided in Supporting Information S1: Figure 2. On all plots, the *x*‐axis represents the normalized treatment duration (1 indicates completion of all 24 scheduled doses), while the *y*‐axis shows the total normalized dose received (1 indicates full‐dose administration of 15 mg for all 24 cycles). Point shapes indicate disease progression. In **(A)**, color reflects hematologic toxicity; in **(B)**, it reflects the number of recorded data points. ASCT, autologous stem cell transplantation; TTP, time to progression.

Meanwhile, clusters BM_B and BM_D show no significant difference between treatment arms, indicating that MRD dynamics in these clusters are inherently favorable or unfavorable, regardless of therapy. All BM and PB clusters were analyzed exclusively for patients in the experimental arm to assess the therapy's impact. Various factors were considered, including treatment duration, lenalidomide dosage, and associated hematological toxicity. The scatter plots in Figure 3A illustrate the distribution of patients based on treatment duration and intensity, along with relapse status (depicted as circles for no event and triangles for relapse) and toxicity levels (represented by color variations). Overall, patients with favorable MRD kinetics who received maintenance lenalidomide had a better prognosis. These findings remained consistent across different time points measured after ASCT (see Supporting Information S1: Figure 3B).

#### MRD classification: Evaluating the accuracy and robustness

To assess the accuracy and robustness of MRD classification, we analyzed both BM and PB data from FIL‐MCL0208 using cluster assignments generated by the CONNECTOR framework. The analysis focuses on discriminative time points that most effectively distinguish between cluster memberships, thereby evaluating the stability and transferability of the clustering outcomes. Moreover, more attention is given to the generalizability of the model by assessing the minimum number of time points required to maintain classification accuracy, the effect of alternating between BM and PB data on cluster assignment, and the contribution of specific time points to accurate classification. The methodological details and results are provided in the Supporting Information S1: Section 2.1.

### Model validation in the *MCL Younger* series

We analyzed MRD longitudinal measurements from the independent “MCL Younger” trial to assess whether predictive models generated using the FIL‐MCL0208 cohort can capture MRD dynamics associated with disease progression and remain generalizable to other datasets. The MCL Younger trial (ClinicalTrials.gov identifier: NCT00209222) is a multicenter, open‐label, parallel‐group, randomized Phase III trial conducted in 128 centers in Belgium, France, Germany, and Poland. The trial enrolled 497 patients aged 65 years or younger with untreated Stage II–IV MCL and suitable for ASCT.^5^, ^8^ The dataset comprises MRD longitudinal measurements from both BM and PB tissues. The data are fed to the *model abstraction* phase of the workflow, together with the estimated predictive model obtained from the FIL‐MCL0208 dataset processed through the *predictive model definition* phase (Supporting Information S1: Figure 5), BM MRD data with at least three time points (median 4, min 3, max 9) were available in 70 patients. Of those, 47 are classified as favorable MRD kinetics (36 in BM_A and 11 in BM_B), and 17 patients are classified as unfavorable MRD kinetics (16 in BM_C and 1 in BM_D). The TTP HR of patients in the unfavorable MRD cluster, BM_C and BM_D, is 2.26 (CI 95% 1.55–3.30). The Kaplan–Meier curves revealed that the median TTP is longer than the study duration for BM_A and BM_B, while it is equal to 37 months for BM_C, see Supporting Information S1: Figure 5. The estimated survival functions for the three CONNECTOR clusters are significantly different (P < 0.0001). Six patients could not be classified. The unclassified group of longitudinal measurements is characterized by a membership probability distribution with (i) maximal assignment probability lower than a specific cut‐off (in this case set to 0.6) and (ii) an entropy value greater than one.

PB MRD data associated with at least four time points (median 7, min 4, max 15) were available in 182 patients. The clusters representing favorable MRD kinetics, PB_A and PB_B, included 145 patients: 121 in PB_A and 24 in PB_B. Among these, 37% and 42% of patients relapsed in PB_A and PB_B, respectively. In contrast, the clusters representing unfavorable MRD kinetics, PB_C and PB_D, comprised 33 patients: 18 in PB_C and 15 in PB_D. In these clusters, the majority of patients experienced relapse, with relapse rates of 95% in PB_C and 100% in PB_D. Survival analysis revealed a significant separation of the Kaplan–Meier curves among the four PB clusters (P < 0.0001). The median TTP for PB_B exceeded the study duration and reached 108 months. In PB_C, the median TTP was 46 months, while in PB_D, the median TTP was 28 months, see Supporting Information S1: Figure 5. The TTP HR of patients in the unfavorable MRD cluster is 8.47 (CI 95% 7.04– 10.19).

MRD longitudinal measurements can be classified by defining specific landmark points to truncate the data. This allows the prediction of MRD kinetics clusters based solely on MRD values measured up to the chosen landmark point. Hence, a landmark analysis was performed to evaluate the association between cluster membership at the selected landmark points and the TTP for patients who had not yet relapsed by those landmark points. The MRD trajectories were classified by setting landmark points at 6‐month intervals from the randomization date.

**Figure 4:**
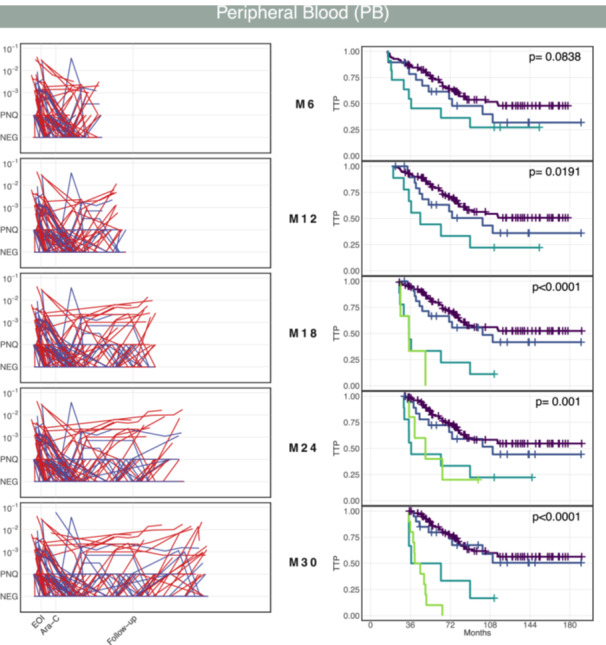
**Landmark analysis of mantle cell lymphoma (MCL) Younger trial.** The first column shows bone marrow (PB) minimal residual disease (MRD) data truncated at the selected landmark points (M6, M12, M18, M24, and M30). The accompanying Kaplan–Meier curves depict the time‐to‐progression (TTP) distribution for all patients, stratified by MCL predictive model classifications obtained at each landmark point. Log‐rank test results are displayed in all Kaplan–Meier plots. NEG, negative; PNQ, positive not quantifiable.

Figure 4 presents the results for MRD data from PB samples. The first column displays the MRD longitudinal measurements truncated at the selected landmark point. On the same row, the Kaplan–Meier curves illustrate the TTP survival distribution across all patients, stratified according to MCL predictive model classifications at each landmark point. The log‐rank test reported in all Kaplan–Meier plots associated with a given landmark point revealed the statistically significant discrimination power of the MCL predictive models. The landmark analysis has also been performed on BM MRD longitudinal measurements, and the results are illustrated in Supporting Information S1: Figure 6.

Anticipating the landmark would be highly desirable to evaluate whether MRD‐based stratification could inform clinical decision‐making at a time when therapeutic interventions remain feasible. However, in the present study, earlier landmarking is not feasible due to constraints inherent to the selected datasets, as patient randomization occurs at diagnosis. In the younger MCL cohort, the definition of an earlier landmark is further constrained by the availability of early MRD measurements that can realistically be used in clinical practice. As a result, the timing of landmarking in this study reflects the clinical feasibility of MRD assessment in the validation cohort. Landmarking performed considerably after the MRD determination window raises concerns about clinical actionability, as therapeutic decisions require results to be available before disease progression or other events occur. To ensure that MRD findings can meaningfully inform treatment adaptation, a study should be specifically designed to collect the first two or three MRD assessments in real time, thereby enabling timely clinical evaluation and potential intervention. Subsequent MRD measurements could then be obtained during follow‐up. Ultimately, enabling earlier clinical actionability would require datasets explicitly structured to allow MRD assessment prior to randomization, aligning molecular stratification with the therapeutic decision‐making window.

## DISCUSSION

This study presents a comprehensive, model‐based workflow for the longitudinal analysis of MRD dynamics in MCL, with the dual aim of automating the discrimination of relapse risk and of supporting subsequent clinical decision‐making.

The development of novel therapies in MCL is rapidly evolving, and alongside these advancements, it is essential to implement analytical approaches capable of extracting the impact of these therapies on disease evolution from available data. Moreover, the issue of immortal time bias is an inherent limitation that must be considered when interpreting treatment effects based on already collected, sequential data. These challenges highlight the need for more sophisticated tools that integrate longitudinal MRD measurements to predict future clinical outcomes. Such tools would also support the design of randomized controlled trials, where data are collected and analyzed in real‐time.

The functional MRD workflow enables the fitting of MRD trajectories through a model with parameters that account for cluster membership and, therefore, population stratification. A predictive classification of new MRD trajectories and a corresponding clinical interpretation are also developed. This integrated strategy leverages temporal modeling to characterize MRD behavior with high resolution and precision. From a methodological perspective, the CONNECTOR tool enabled efficient data analysis and visualization, while the entropy analysis provided insights into the confidence of cluster assignments.

The application of this workflow to MRD data from both BM and PB blood samples within the FIL‐MCL0208 trial demonstrated strong predictive performance. Specifically, the analysis yielded a predictive model with four distinct MRD dynamics, which were subsequently collapsed into two clinically relevant groups: favorable MRD, marked by sustained negativization, and unfavorable MRD, characterized by fluctuating MRD trends. The favorable MRD cluster is associated with low relapse rates, while the unfavorable MRD cluster is related to elevated relapse frequencies. Moreover, Kaplan–Meier analyses confirmed a statistically significant separation of survival functions across the identified clusters, underscoring the discriminative capability of the predictive models.

Validation on an independent cohort—the MCL Younger trial—confirmed the generalizability of the model. Here, stratification patterns remained consistent with data, and separation of survival curves across MRD‐defined clusters was preserved. The landmark analysis further substantiated the discriminative capacity of the model at multiple time points, identifying key time points with the highest discriminatory power. These results illustrated that early prediction of relapse is feasible and clinically actionable and provide a robust basis for patient stratification, particularly in predicting long‐term treatment outcomes.

Interestingly, discriminant analysis identified treatment‐associated intervals—such as early MRD determinations (i.e., post‐RCHOP and post‐ASCT time points) in BM, and late‐phase ones (i.e., M24–M30) in PB—as the most informative time points for MRD assessment. This experimental evidence underscores the tissue‐specific nature of MRD dynamics and holds deep clinical relevance, strongly supporting the value of defining MRD using repeated measurements in PB samples, potentially dispensing the clinician from obtaining data from invasive, “patients‐unfriendly” BM examinations.

Entropy‐based uncertainty quantification revealed that cluster assignment confidence improves significantly with an increasing number of MRD time points. This finding reinforces the need for rich longitudinal sampling to achieve accurate trajectory modeling and risk classification. The analysis also revealed that this predictive MRD classification is independent of the traditional clinical and biological baseline prognostic parameters, further emphasizing its clinical impact. This finding elevates the role of MRD monitoring as a uniquely powerful biomarker in MCL, capable of capturing disease evolution in a way that baseline diagnostic information cannot. We should acknowledge that these data come from two clinical trials that offered conventional chemo‐immunotherapy and ASCT,^8^, ^17^ whereas the current therapeutic scenario in MCL is rapidly evolving toward the incorporation of Bruton's tyrosine kinase inhibitors into first‐line treatment, along with the omission of ASCT.^19^, ^20^, ^21^, ^22^, ^23^ However, promising data on the prognostic impact of MRD, even in these novel settings, have been recently presented at the 18th International Conference on Malignant Lymphoma (ICML) by Khouja and Zinzani. The validation of our computational approach in the Triangle trial^19^ is currently ongoing within the “Multiply” translational research project, funded by the Leukemia & Lymphoma Society (https://multiply.hpc4ai.unito.it/).

From a methodological standpoint, the proposed workflow provides a powerful and user‐friendly framework for analyzing and visualizing longitudinal MRD data. It enables the robust characterization of temporal patterns across patient subgroups, facilitating a deeper understanding of disease dynamics and treatment response. While the methodology is applied here to MRD data from MCL, it is readily scalable to other lymphoma subtypes and clinical settings in which a biologically relevant biomarker is measured longitudinally. Specifically, the TRIANGLE study^19^ provides an ideal clinical trial setting in which patients can be prospectively stratified by risk class. Complementing this, the *MCLexplore* web application offers an interactive platform for users to engage with the predictive models. It allows exploration of both clinical and biological features that define each cluster and supports the classification of new longitudinal data.

This dual approach not only enhances model interpretability but also empowers clinicians and researchers to apply the framework in real‐world settings, ultimately bridging the gap between computational analysis and clinical decision‐making.

From a future perspective, the potential implications of MRD dynamics for defining treatment procedures and stratifying patients with MCL will be significantly enhanced by rapid advances in emerging technologies. This includes the use of deep sequencing to track all evolving tumor clones and the increasing variety of data types collected in clinical settings, such as mutational and gene expression profiles. The development of a comprehensive integrative model that can incorporate all these insights into a unified framework is the next critical step to pursue.

## AUTHOR CONTRIBUTIONS

**Francesca Cordero**: Methodology; conceptualization; writing—original draft; formal analysis; writing—review and editing. **Simone Ferrero**: Conceptualization; writing—original draft; data curation; resources. **Simone Pernice**: Writing—original draft; methodology; software. **Elisa Genuardi**: Formal analysis; data curation; validation. **Daniela Volpatto**: Software; formal analysis; data curation. **Roberta Sirovich**: Software; formal analysis; data curation; methodology; writing—original draft. **Aurora Maria Civita**: Data curation. **Andrea Evangelista**: Formal analysis. **Simone Ragaini**: Data curation. **Alice Di Rocco**: Data curation. **Alessandro Re**: Data curation. **Vittorio Stefoni**: Data curation. **Federica Cavallo**: Data curation. **Carola Boccomini**: Data curation. **Monica Balzarotti**: Data curation. **Vittorio Ruggero Zilioli**: Data curation. **Maria Gomes da Silva**: Data curation. **Luca Arcaini**: Data curation. **Melania Celli**: Data curation. **Gian Maria Zaccaria**: Formal analysis. **Dora Tortarolo**: Formal analysis; data curation. **Marco Beccuti**: Formal analysis; software. **Eva Hoster**: Formal analysis; validation. **Christiane Pott**: Data curation. **Elizabeth Macintyre**: Data curation. **Olivier Hermine**: Resources; funding acquisition. **Martin Dreyling**: Resources; project administration; funding acquisition. **Marco Ladetto**: Funding acquisition; project administration; resources.

## CONFLICT OF INTEREST STATEMENT

S.R. received speaker's honoraria from Roche, BeiGene, and Pierre Fabre. S.F. is a consultant for Janssen, EUSA Pharma, AbbVie, and Sandoz; is on the advisory board of Janssen, EUSA Pharma, Recordati, Incyte, Roche, AstraZeneca, CSL Behring, and Italfarmaco; received speaker's honoraria from Janssen, EUSA Pharma, Recordati, Lilly, BeiGene, Gilead, and Gentili; and received research funding from Gilead, BeiGene, and Morphosys. M.L. has relationships in terms of consultancy, participation in advisory boards, invitation to scientific meetings, institutional research support, and contracts with AbbVie, Acerta, Amgen, ADC Therapeutics, BeiGene, Celgene/BMS, Eusapharma, GSKI, Gentili, Gilead/Kite, Novartis, Incyte, J&J, Jazz, Lilly, Regeneron, Roche, and Sandoz; he has non‐financial interests as PI or strategic investigator in studies supported by Celgene, J&J, BeiGene, and ADC Therapeutics.

## ETHICS STATEMENT

The (FIL)‐MCL0208 is registered with EudraCT (2009‐012807‐25) and ClinicalTrials.gov (NCT02354313, first registration on 03/02/2015). The clinical trial was approved by the Ethical Committees of all the enrolling Centers. All patients provided written informed consent for the use of their biological samples for research purposes, in accordance with the Institutional Review Board's requirements and Helsinki's declaration.

## FUNDING

Supported by a grant from The Leukemia & Lymphoma Society (LLS grant no. MCL 7005‐24); Progetto di Ricerca Sanitaria Finalizzata 2021 (RF‐2021‐12371972, CUP G13C21001540001), Torino, Italy, and Alessandria, Italy; S.P. was supported by grants from “MineClust: Knowledge extraction from multivariate longitudinal data in precision medicine” (PERS_CRT_24_01) project funded by CRT foundation (PI Simone Pernice). Open access publishing facilitated by Universita degli Studi di Torino, as part of the Wiley – CRUI‐CARE agreement.

## Supporting information

Supporting Information.

Supporting Information.

## Data Availability

The data that support the findings of this study are available in the supplementary material of this article.
