# Supplementary material for: Dissecting minimal residual disease dynamics to improve outcome prediction in mantle cell lymphoma: Data from the Fondazione Italiana Linfomi (FIL)‐MCL0208 clinical trial
Source: Hemasphere. 2026 Apr 30;10(5):e70375. doi: 10.1002/hem3.70375 (PMC13129670; doi:10.1002/hem3.70375)
Supplement: Supplementary file 1 — Supporting Information. [file HEM3-10-e70375-s001.docx]

# **Supplementary Information**

**S1. Material and Methods**

## **S1.1 Details on the functional MRD workflow**

The following paragraphs provide a detailed description of the predictive model definition and model abstraction phases of the workflow shown in Figure S1.


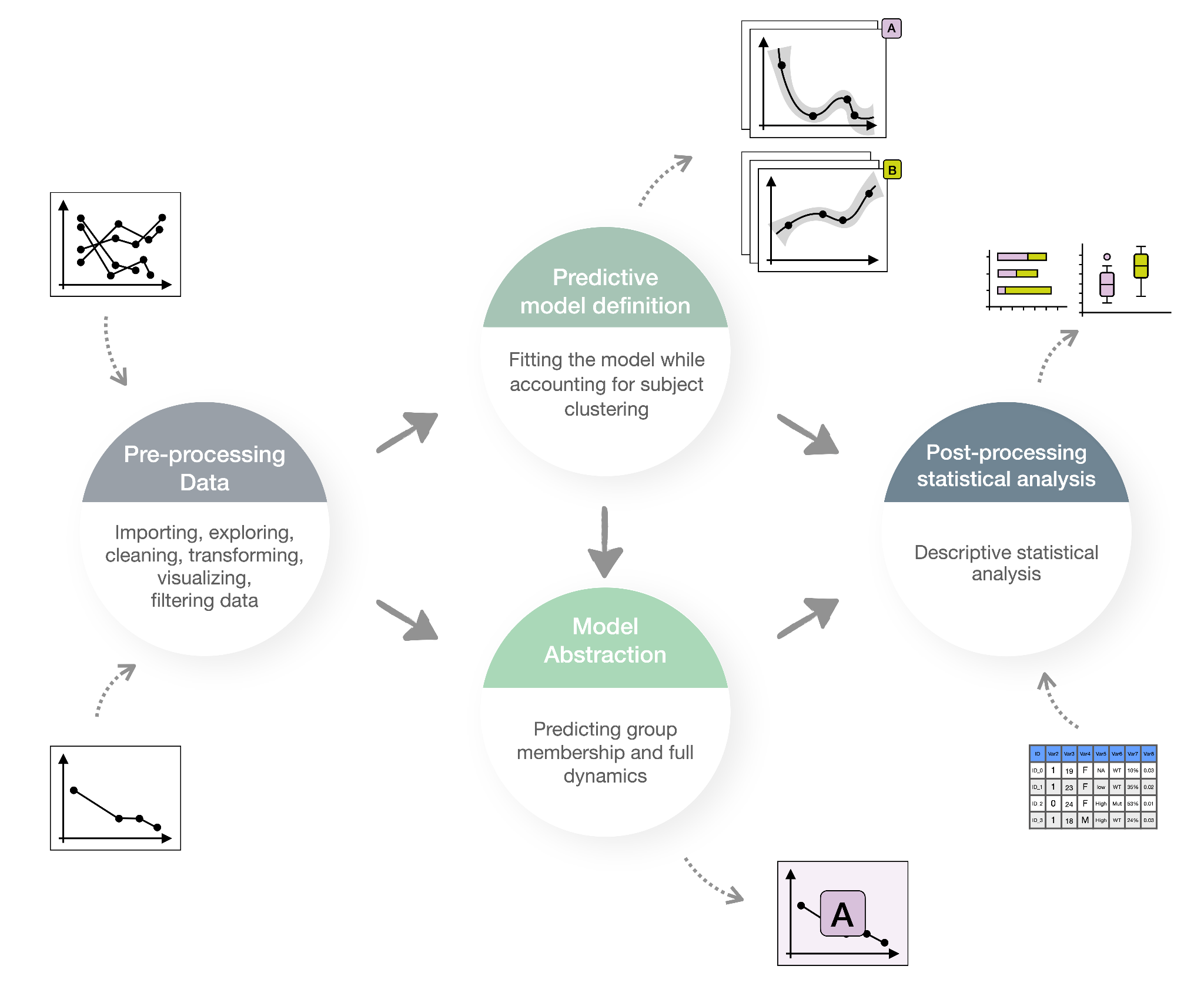


*Figure S1: The functional MRD workflow. MRD longitudinal data are given in input to the tool; CONNECTOR automatically clusterizes the curves defining a model, then new curves can be assigned to the most appropriate cluster among those defined by the model. The clusterization and/or classification can then be assessed against other possible clinical stratifications.*

**Predictive model definition** CONNECTOR is a tool for the unsupervised analysis of longitudinal data, it can process any sample consisting of measurements collected sequentially over time. For a complete description, please refer to [16]. CONNECTOR is built on the model-based approach for clustering functional data presented in [17], which is particularly effective when observations are sparse and irregularly spaced in time. The CONNECTOR tool guides the user in performing a functional clustering task composed of two main parts: selecting free parameters and learning model parameters, including the cluster membership probabilities for each curve, which determine their assignment to the respective classes.

The free parameters are the dimension p of the spline basis, used to reconstruct the curves at the unmeasured time points, and the number of clusters G. The choice for p is supported by two plots: the first represents the 10-fold cross-validated log-likelihood values for different options of the spline basis dimension, and the second displays the corresponding knot positions on the time axis. A good value for p is obtained at the elbow for the most significant log-likelihood values and well-distributed knot points along the observation time interval. To help with the number of cluster choices, CONNECTOR returns two plots. The first one contains the violin plots of the functional Davies and Bouldin (fDB) indexes, introduced in [16], for repeated clusterings with different numbers of groups G. The smaller the fDB values are, the better the final clustering is. As the algorithm is randomly initialized, more repetitions are run. The second plot represents the violin plots of the total tightness (TT) values, introduced in [16], for repeated clusterings with different numbers of groups G. The smaller the TT values are, the better the final clustering is, while remembering that TT tends to zero as the number of clusters increases and each curve clusters to itself. Hence, the optimal number of clusters G is obtained as the value that minimizes the fDB indexes at the elbow for the smallest TT values.

Once the free parameters are set, CONNECTOR learns the model parameters through an EM algorithm in which cluster memberships are considered missing data. Hence, the output is composed of the cluster assignments for each curve (and a plot of the curves separated into the clusters), the model parameters (which include the fitting for each curve with confidence bands), and the discriminant functions (a set of curves that identify the time points of maximum discrimination between clusters).

**Model abstraction** In this paper, we present a newly integrated module in CONNECTOR designed for functional classification, aimed at classifying previously unseen curves into one of the $G$ possible groups. This new module of CONNECTOR mainly depends on the module that estimates the predictive model. Indeed, the clustering algorithm within CONNECTOR is carried out by estimating a predictive model, where the probabilities of group membership are treated as model parameters. Under the estimated predictive model, the joint law of the observations of the $i$-th selected curve, given the class membership, is explicitly known. Thus, the model can classify new longitudinal measurements, not involved in the model estimation, into one of the $G$ possible clusters by explicitly calculating the posterior group membership probabilities (as the new curve is observed) using Bayes’ formula and the likelihood of the curve given its group membership. Let us now describe in details the functional clustering model and the classification procedure. Let $Y_{i}$ be the vector of observed values of the $i$-th subject at times $t_{i_{1}},...,t_{i_{ni}}$. Individual curves are modeled using basis functions, particularly cubic splines. The spline coefficients are treated with a random effect model and furthermore rewritten with a lower-dimensional representation of the curves with means in a restricted subspace, see [17] for a detailed description. The functional predictive clustering model can be written as

$$Y_{i}=S_{i}\cdot\left( \lambda_{0}+\Lambda\alpha_{z_{i}}+\gamma_{i} \right)+\epsilon_{i}, i=1, \ldots,n$$

$$\epsilon_{i}\sim N\left( 0, \sigma^{2}I \right), \gamma_{i}\sim N(0,\Gamma)$$

where $S_{i}=\left( s\left( t_{i_{1}} \right), \ldots, s\left( t_{i_{ni}} \right) \right)^{T}$ is the spline basis matrix for the $i$*−*th curve, $\lambda_{0}$ and $\alpha_{k}$ are *p−* and *h−* dimensional vectors, $\Lambda$ is a $\left( p,h \right)$ matrix and $h\leq$ min($p,G-1$), where $G$ denotes the number of clusters. Thus, $h$ represents the reduced dimension of the mean space (the fixed effect part of the model), while $\gamma_{i}$ is the random effect vector for the $i$-th selected individual. The $z_{i}$ denotes the unknown cluster membership, treated as missing data through the estimation procedure, which is carried out using an EM algorithm.

Hence, once the model has been trained on the dataset, the parameters $\lambda_{0}, \Lambda, \alpha_{k}, \Gamma$ and $\sigma^{2}$ have been estimated.

Under the model (1), the joint law of the observations of the $i$-th selected curve, given the class membership is $k$, is known

$$Y_{i}|class=k\sim N(S_{i}\cdot\left( \lambda_{0}+\Lambda\alpha_{k} \right), S_{i}\cdot\Gamma\cdot S_{i}^{T}+\sigma^{2}I)$$

Hence, the model can be used to classify new curves $Y^{new}$ which have not been involved in the model estimation, into one of the $G$ possible clusters. The posterior probabilities $P\left( class=k \right| Y^{new})$ can be calculated using the Bayes formula

$$P\left( class=k \right| Y^{new})= \frac{f\left( Y^{new} \right| class=k)\cdot\pi_{k}}{\sum_{j=1}^{G} f\left( Y^{new} \right| class=j)\cdot\pi_{j}}$$

Where $\pi_{j}$ is the prior probability of being class $j$ and $f$ is the density of a multivariate Gaussian distribution with mean vector $S_{new}\cdot\left( \lambda_{0}+\Lambda\alpha_{k} \right)$ and covariance matrix $S_{new}\cdot\Gamma\cdot S_{new}^{T}+\sigma^{2}I$. The posterior probabilities are given in eq. (2) and are calculated for any $k.$

Once the posterior probabilities have been calculated for any subject, the observations are assigned to the class with maximal membership probability. However, the whole posterior distribution is informative, and we decided to include such information in the discussion. A measure of the strength of the class assignment can be extracted as the Shannon entropy of the posterior distribution, defined as

$$H\left( X \right)= - \sum_{x} P\left( X=x \right)\cdot\log P\left( X=x \right), x\in Im(X)$$

for a given random variable with the discrete image set $Im(X$). If we set the class values as positive integers (for example $1, 2, \ldots,G$), the distribution with maximal entropy is the uniformly discrete, where each membership probability equals $1/G$. This is the worst-case scenario for the class assignment, as the posterior distribution cannot clearly indicate a *preferable* class for the observation. Hence, large entropy values are associated with weak class assignments, while small entropy values reveal strong class assignments. Building on this concept, we add to the $G$ groups predicted by the functional clustering model an extra class to accommodate curves that cannot be classified with high confidence. We refer to this group as the group of *unclassified* curves, characterized by a membership probability distribution with (i) maximal assignment probability lower than a specific cut-off (for example 0.6), and (ii) an entropy value greater than one.

**S1.2 Details on the post-processing statistical analyses**

The methodologies implemented to explore results include:

**Landmark Analysis -** Traditional survival analysis was performed to explore the survival characteristics (progression of the disease) of patients with MRD curves grouped into the CONNECTOR clusters. However, a shift to a landmark analysis was essential to evaluate the survival outcomes of patients grouped by clusters derived from MRD curves observed at different time points (restricted to the overall study period). The landmark analysis considers the group membership covariate, which is time-dependent in this case, calculated as follows: at each landmark time, the CONNECTOR clusters were estimated based on the MRD curves within a restricted time interval, where the endpoint corresponds to the given landmark, and the cluster memberships were consequently assigned. Thus, a survival analysis was performed on the at-risk subjects at the landmark point.

**Descriptive Statistics -** We compared categorical variables with Chi-Square or Fisher’s exact test. Fisher’s test was applied when at least one of the elements of the contingency table contained fewer than five elements or when the contingency table was 2x2. To test independence between groups created according to a qualitative variable of a quantitative observable, whenever the qualitative variable consisted of two groups, the following tests were conducted: the Kruskal-Wallis test to determine if there were significant differences in the medians between the groups, the T-test (if the Shapiro test indicated normal distribution or if each group contained more than 50 data points) or the Wilcoxon-Mann-Whitney test (otherwise) to assess the equality of means, the F-test (if the Shapiro test indicated normal distribution) or Levene’s test (otherwise) to evaluate the equality of variances, the Kolmogorov-Smirnov test when the sample size was sufficiently large. When there were more than two groups, the following tests were performed: the Kruskal-Wallis test to assess differences in medians across the groups, the ANOVA test to evaluate the equality of means, Bartlett’s test (if the Shapiro test indicated normal distribution) or Levene’s test (otherwise) to assess the equality of variances.

### **Chord diagram -** A chord diagram is a circular visualization technique used to represent relationships among categories of data points. A chord diagram arranges data along the circumference of a circle, with connections and interactions displayed as arcs or ribbons, called chords, that span across the circle. These chords’ thickness, color, and curvature can represent different aspects of the relationships, such as magnitude or type of interaction. Colors differentiate between categories, enhance contrast, or indicate specific data characteristics. This paper used a chord diagram to compare different patient clusterings, mapping the concordance between the groupings. Hence, chords highlighted the relationships between clusters from others analyses, showing both matches (chords that map similar clusters) and discrepancies (chords that map dissimilar clusters).

**MCL Explorer - Shiny Application -** We designed an interactive application to analyze, inspect, and visualize clustering and classification results reported in the paper. The application is available at <https://github.com/qBioTurin/MCLexplorer>, and is built with the Shiny package, providing a structured, user-friendly interface in R. The dashboard, titled MCL Explorer, features a navigation menu with three primary sections. The first section, “*Clustering Exploration*”, allows users to delve into clustering results through entropy analysis. The second section, “*Classification Exploration*”, is dedicated to exploring classification outcomes, specifically from a dataset known as the MCL Younger trial. The last section, “*Classify your data*”, allows users to upload their datasets and perform classifications on them. This section is divided into two subsections: “Upload” where users can upload and preview their data, and “*Classification*” where they can run the classification.

*Clustering Exploration.* In the first section, users can select the tissue type (PB or BM) and visualise the clustering results of the MCL0208 dataset. Specifically, five possible analyses can be performed: (1) the clustering visualisation, (2) the clinical assessment, (3) the survival analysis, (4) the cluster entropy analysis and (4) the data inspection through an interactive table. The clustering visualization is presented through two graphics. The first is a simple line plot where the MRD curves are separated by clustering membership and colored according to their respective entropy values. The second is a scatter plot that displays the entropy values for each curve based on the length of the curve (number of observations) and the clustering membership. The clinical assessment enables users to assess the statistical significance of cluster subdivisions by analyzing variables such as pharmacogenomics, gene mutations, and anonymized patient metadata. Meanwhile, the survival analysis generates a Kaplan-Meier plot that stratifies patients by cluster, merged cluster, or treatment group. Additionally, users can filter plots based on entropy and curve length. This filtering step also affects the final visualization, which focuses on inspecting the curve fittings obtained from CONNECTOR. Finally, an intuitive and interactive table of the clustered data is available for inspection, providing a comprehensive overview of the analyzed cohort.

*Classification Exploration.* The second section is designed for analyzing the “Younger data” classification results. Users can choose tissue types, truncate curves, and select variables to color plots. The tab includes panels for displaying line plots, performing classification (see Section “*Details on the workflow phases, Model abstraction*”), displaying the survival analysis considering the cluster classification, and landmark analysis (see Section “*Details on the workflow phases, Post-processing statistical analysis*”).

*Classify your data.* The last section provides a comprehensive interface for users to upload their data and run classifications considering MCL0208 trial clusters. In the *Upload* subsection, users can upload Excel and TXT files, preview the data, and prepare it for classification. The *Classification* subsection provides functionality similar to the *Classification Exploration* section, enabling users to select features, adjust parameters, and visualize classification results through various plots.

**Standardization of treatment quantification and duration**

Formal mathematical derivation of normalized treatment duration, normalized hematological toxicity, and normalized drug quantity, corresponding to the metrics used in Figure 3. Treatment duration is expressed as the fraction of the planned 24 doses that a patient actually received. Drug quantity is expressed as the proportion of the total expected amount (24 doses of 15 mg each) that was administered. Hematological toxicity is described as the proportion of treatment cycles in which toxicity was observed relative to the number of doses the patient received. A graphical representation of the mathematical derivation is reported in Figure S2.


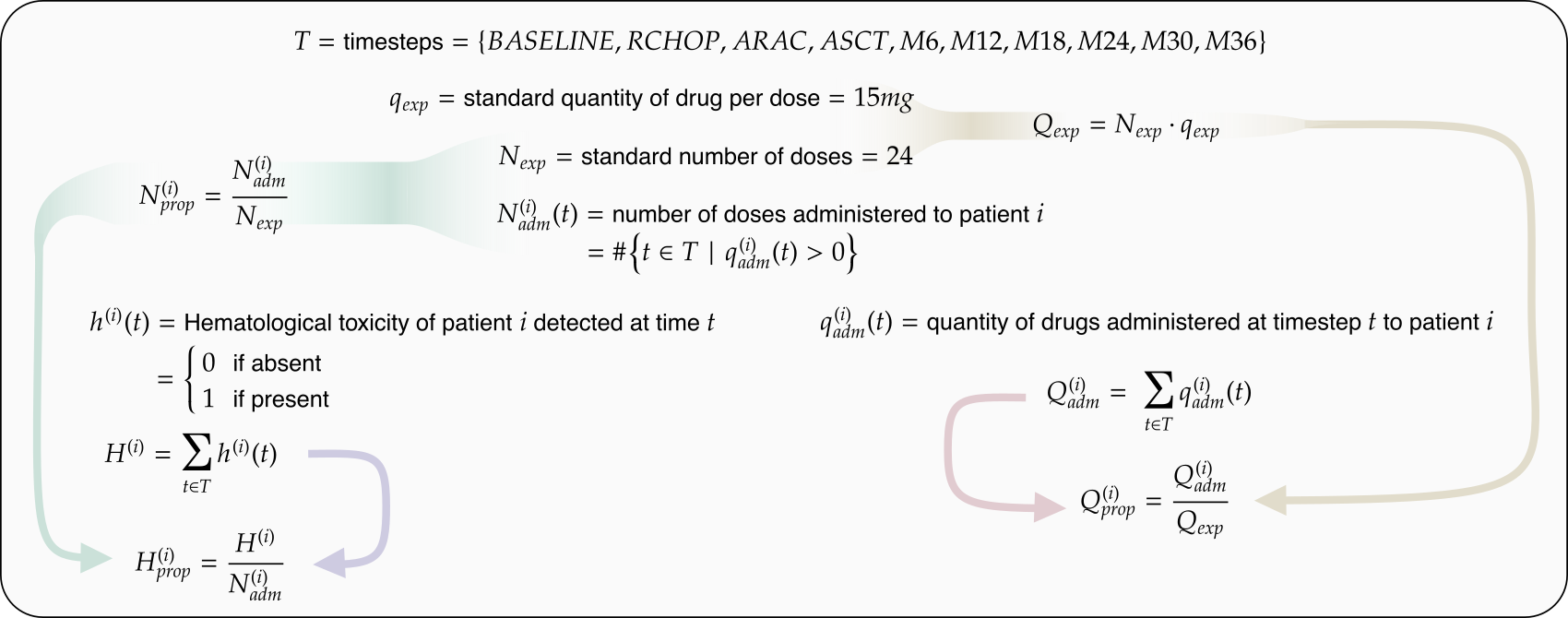


*Figure S2: Formal mathematical derivation of the normalized treatment duration*

## **S1.3 The FIL-MCL0208 Cohort**

Biological variables of FIL-MCL0208 trial**.** Clinical and biological data, other than MRD (mutations [19] and pharmacogenomics [20], Table S1, were generated in the context of dedicated ancillary studies of the FIL MCL0208 trials. For methodological details, please see the respective publications.

**
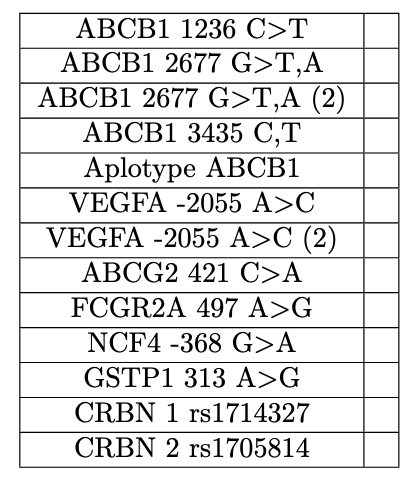
**

*Table S1: List of pharmacogenomics mutations*

## **Clinical baseline and longitudinal characteristics of the FIL-MCL0208 series.** In this paper, 117 patients with a minimum of three recorded time points in BM tissue (median: 6; range: 3–9) and 95 patients with at least four recorded time points in PB tissue (median: 7; range: 4–9) have been selected. These patients are a subset of the complete FIL-MCL0208 cohort. In Table S2, we report the distribution of the main clinical features of the entire cohort of patients relative to the subset used in this paper.


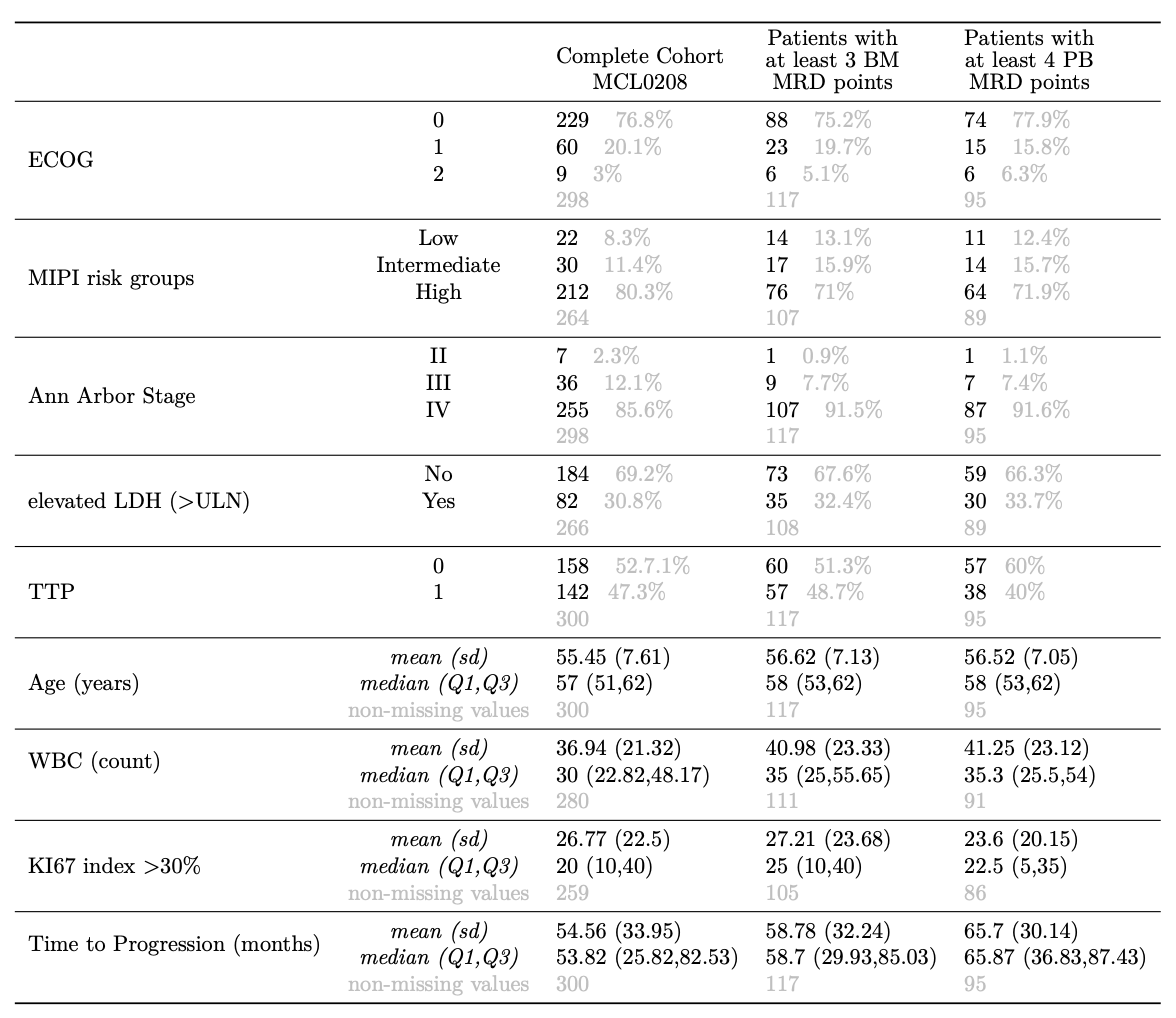


*Table S2: Clinical features*

**Data pre-processing.** Since the baseline measurement is not counted as a time point, the recorded time points begin from the R-CHOP treatment phase. The RQ PCR values used in the analysis were transformed as log in base 10. All patients who meet the minimum-observation constraints outlined above are included in the analysis, even if all their RQ-PCR MRD values are below 10*^−^*^8^. The time points are not standardized across trial phases; instead, the original recorded dates are used. For clarity, in all figures presented in this paper, RQ-PCR values are displayed using their original numerical values. However, the y-axis labels indicate PNQ and NEG, representing biologically relevant concentration thresholds. The x-axis shows the time steps defined in the trial, while the box plots show the distribution of the recorded dates.

**S2. Results**

**S2.1 MRD Classification: Evaluating the Accuracy and Robustness**

## Specifically, we applied a classification process that assigns test samples to the cluster with the highest posterior probability of membership. The longitudinal data from FIL MCL0208 trial were used to define the predictive models trained on BM and to classify the test data composed of various combinations of MRD measurements from both BM and PB. The primary aim was to investigate whether a patient cluster membership—initially determined from full BM MRD trajectories—could be reliably recovered when only partial or mixed tissue data were used.

## This analysis explores the model generalizability by addressing several key questions: (i) What is the minimum number of BM-derived time points needed to maintain classification accuracy? (ii) How does alternating between BM and PB data affect cluster assignment? (iii) How critical are specific time points to accurate classification?

To answer these questions, we carried out five validation experiments. The rationale under the experiments is the following: the functional clustering model (1) is trained on the bone marrow tissue MRD longitudinal measurements included in the FIL-MCL0208 trial, and then five different new MRD longitudinal datasets are classified, according to the posterior probabilities given in Eq. (2), into the class with maximal membership probability. Hence, the model is estimated only once, while the classification task is repeated for the five different validation datasets. The validation datasets are generated to test the stability and robustness of the functional clustering model. The five experiments are summarized in Table S1 and described hereafter:

- **Experiment #1:** The validation dataset comprises the peripheral blood tissue MRD longitudinal measurements in the FIL-MCL0208 trial. Hence, the question addressed here is whether the predictive model estimated from BM longitudinal measurements of patients can be used to make predictions based on longitudinal measurements taken from a different tissue, namely PB.
- **Experiment #2:** The validation dataset comprises synthetic MRD longitudinal measurements obtained by a random combination of timed values from the BM and PB observations. Hence, the MRD points from both tissues make up new longitudinal measurements with mixed values. This dataset is included to test whether the clustering model is robust to MRD longitudinal measurements obtained from a mixture of tissues. The synthetic dataset contains subjects and time points similar to the original dataset.
- **Experiment #3:** In [6], the authors suggest M12 to be a highly informative time point for the disease evolution; hence, the third synthetic dataset is composed of MRD longitudinal measurements with the value at M12 extracted from the BM tissue observations, and the remaining time points extracted from PB tissue observations.
- **Experiment #4:** Following the same reasoning that guided Experiment #4 and aiming to assess the influence of the initial time points, the fifth synthetic dataset comprises MRD longitudinal measurements with RCHOP, ARAC, and ASCT values from BM tissue and the remaining time points from PB.
- **Experiment #5:** CONNECTOR returns the discriminant functions plot, introduced in [17]. As illustrated in [16], those functions identify the time points of maximum discrimination between clusters.

*
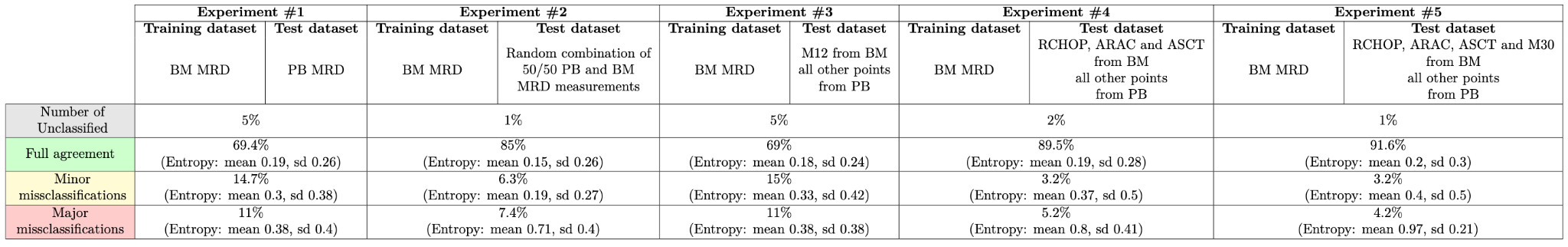
*

*Table S3: Overview of the five experiments with associated evaluation metrics.*

For each experiment, the performance of the predicted classification is evaluated using the following metrics. The first is the proportion of subjects classified in the same MRD CONNECTOR clusters as in the training BM MRD dataset. Those are the best cases, where the validation dataset is in complete agreement with the trained model. The second and third metrics are designed to distinguish the severity of the error into minor and major misclassifications. The proportion of subjects classified in different MRD clusters but within the same risk category (favorable or unfavorable) is calculated and considered a minor misclassification. Analogously, the proportions of subjects classified into different MRD CONNECTOR clusters and risk categories (favorable or unfavorable) are calculated, reflecting the major misclassifications. Finally, for each metric, the entropy of the membership probability distribution is indicated. This quantity indicates a strong cluster assignment when the entropy is small and a weak cluster assignment when the entropy is large. The results are collected in Table S3.

The agreement of the crossed-classification of PB longitudinal measurements to the BM clusters is very high: 87% of the subjects are classified in the same risk category, with 70.5% classified in the same cluster, see Table S1-Experiment #1. The performance is further enhanced when the test synthetic dataset is constructed by combining tissue measurements. In Experiment #2, where the proportion of measurements from the two tissues is 50/50, the agreement increases to 92%, with a full agreement for 86% of the subjects, see Table. S1-Experiment #2. The results of Experiments #4 and #5 are particularly noteworthy, as they achieve the highest concordance between the train and test groups, confirming the significance of the specified time points in predicting the disease evolution. The performance achieved by including only the BM measure at time point M12 in the test set does not differ from that of the full PB test set. Hence, the high discriminative power of that time point, suggested in [6], is not confirmed by the workflow of analysis presented here.

Based on the results presented here, it can be concluded that the MRD CONNECTOR clusters are stable and robust under perturbations of the measurements across tissues.

## **S2.2 CONNECTOR analysis details and Visualization**

### **CONNECTOR reports on FIL-MCL0208 analysis** A total of 117 patients with at least three recorded time points in BM tissue (median: 6; range: 3–9) and 95 patients with at least four recorded time points in PB tissue (median: 7; range: 4–9) were included in the analysis.


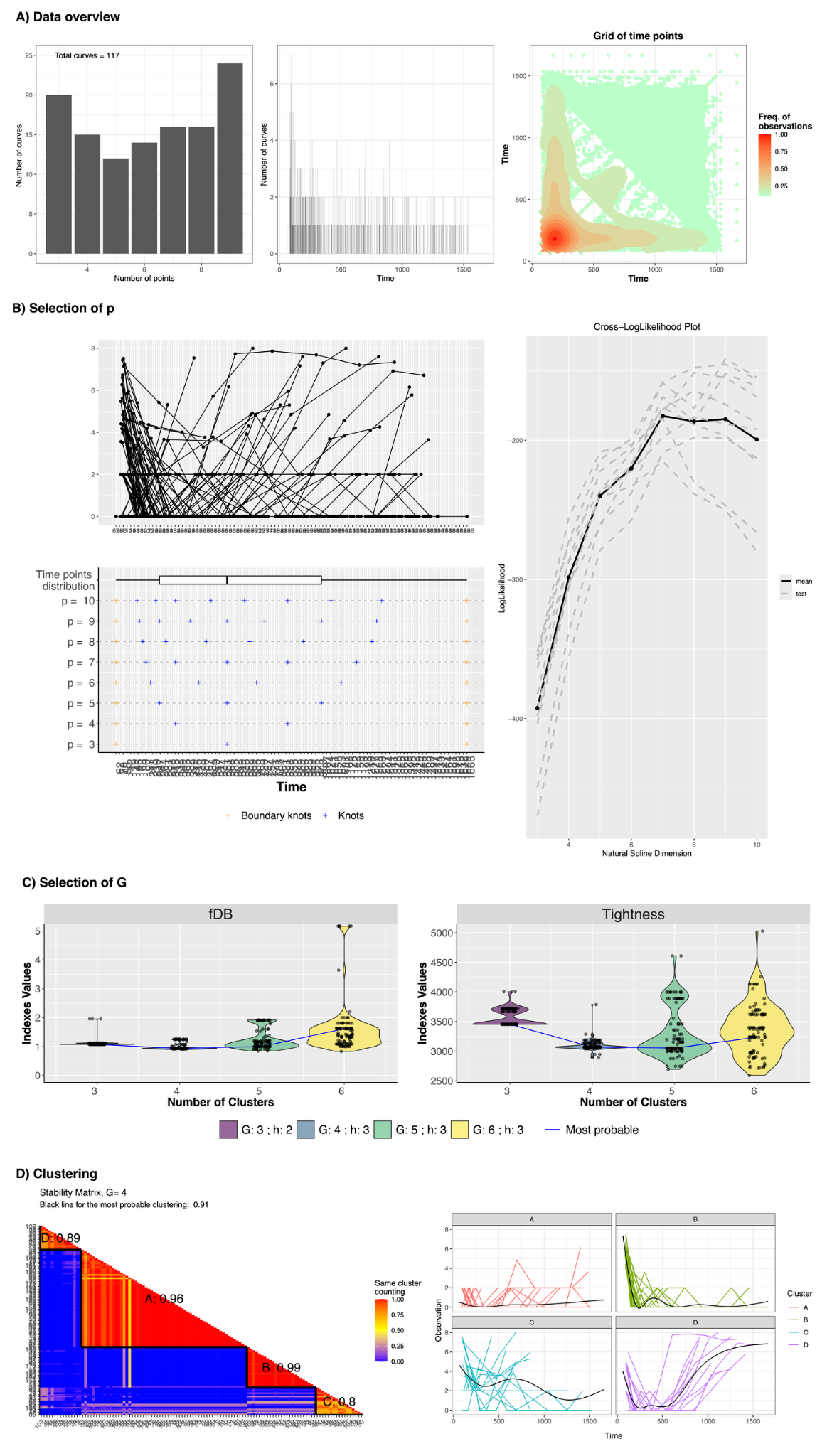


*Figure S3: CONNECTOR report obtained by the analysis of BM MDR data of FIL-MCL0208 cohort*


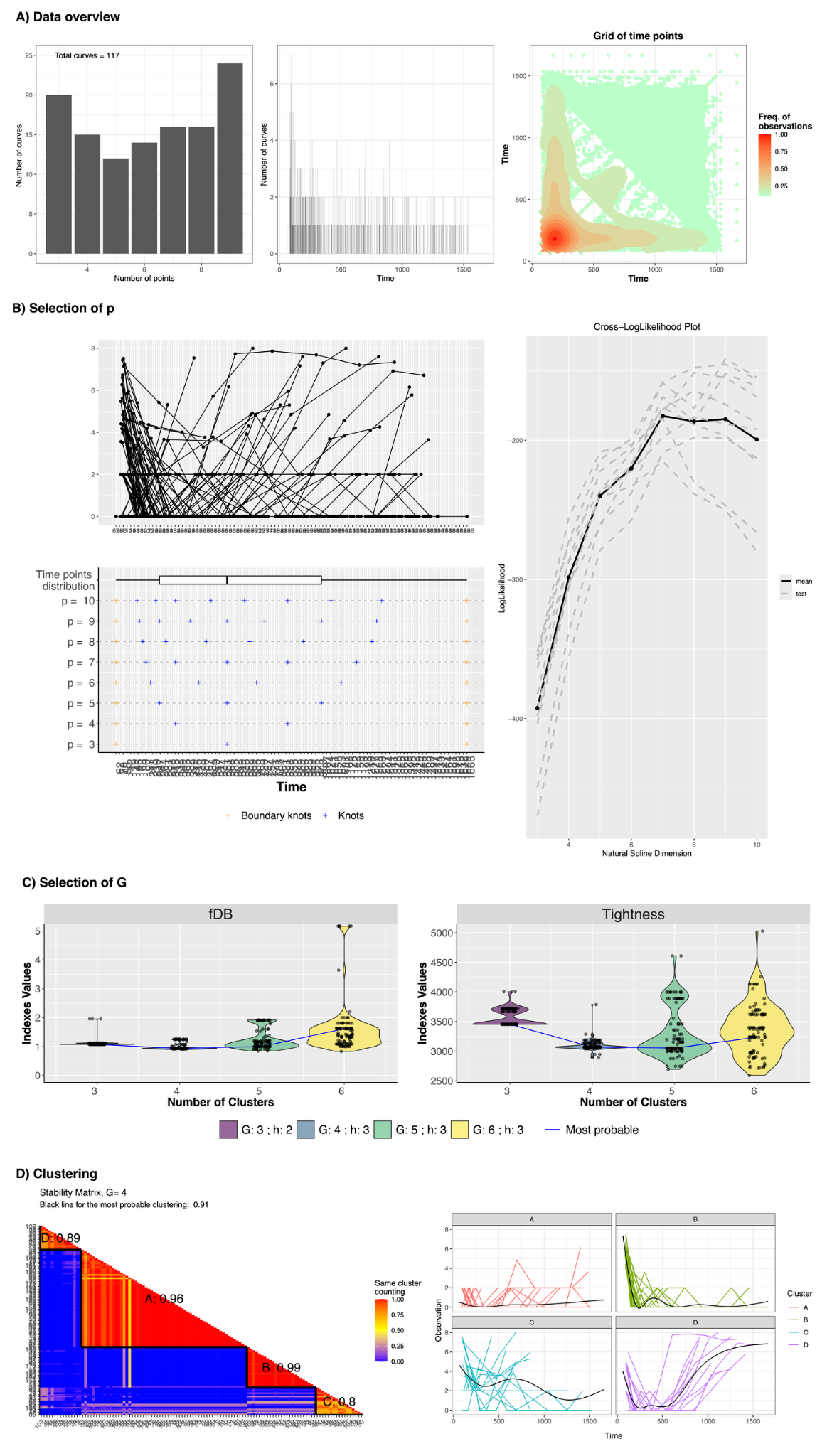


*Figure S4: CONNECTOR report obtained by the analysis of PB MDR data of FIL-MCL0208 cohort*

Figures S3 and S4 present the complete results from the CONNECTOR analysis for BM and PB tissues, respectively. The reports are structured into four sections: (i) Data Overview provides an initial dataset summary. It includes two histograms illustrating the distribution of time points across the number of curves and a time grid to visualize the sparsity of collected time points. (ii) Selection of p. Three plots are designed to assist in selecting the dimension of the spline basis vector. These plots guide the user in determining the most appropriate spline complexity for the analysis. (iii) Selection of G focuses on selecting the number of clusters. It provides visualizations to support the determination of the optimal cluster count for grouping patient trajectories. Finally, (iv) Clustering displays the clustering results, including the stability matrix, which evaluates cluster robustness, and plots of patient dynamics grouped according to the identified clusters. The dimension of the spline basis vector (p) is 7 for BM and 7 for PB. The optimal number of clusters, as suggested by fDB, tightness, and clustering stability, is four for both tissue types.

### **S2.3 The model abstraction on younger MCL cohort**

The younger MCL cohort has been used to verify the generalization ability of the MCL predictive model. The Model abstraction phase is executed by inputting the MCL predictive models obtained from FILMCL0208 cohort and the never-seen MRD curve of the MCL younger cohort. The results are reported in Figure S5.

### **
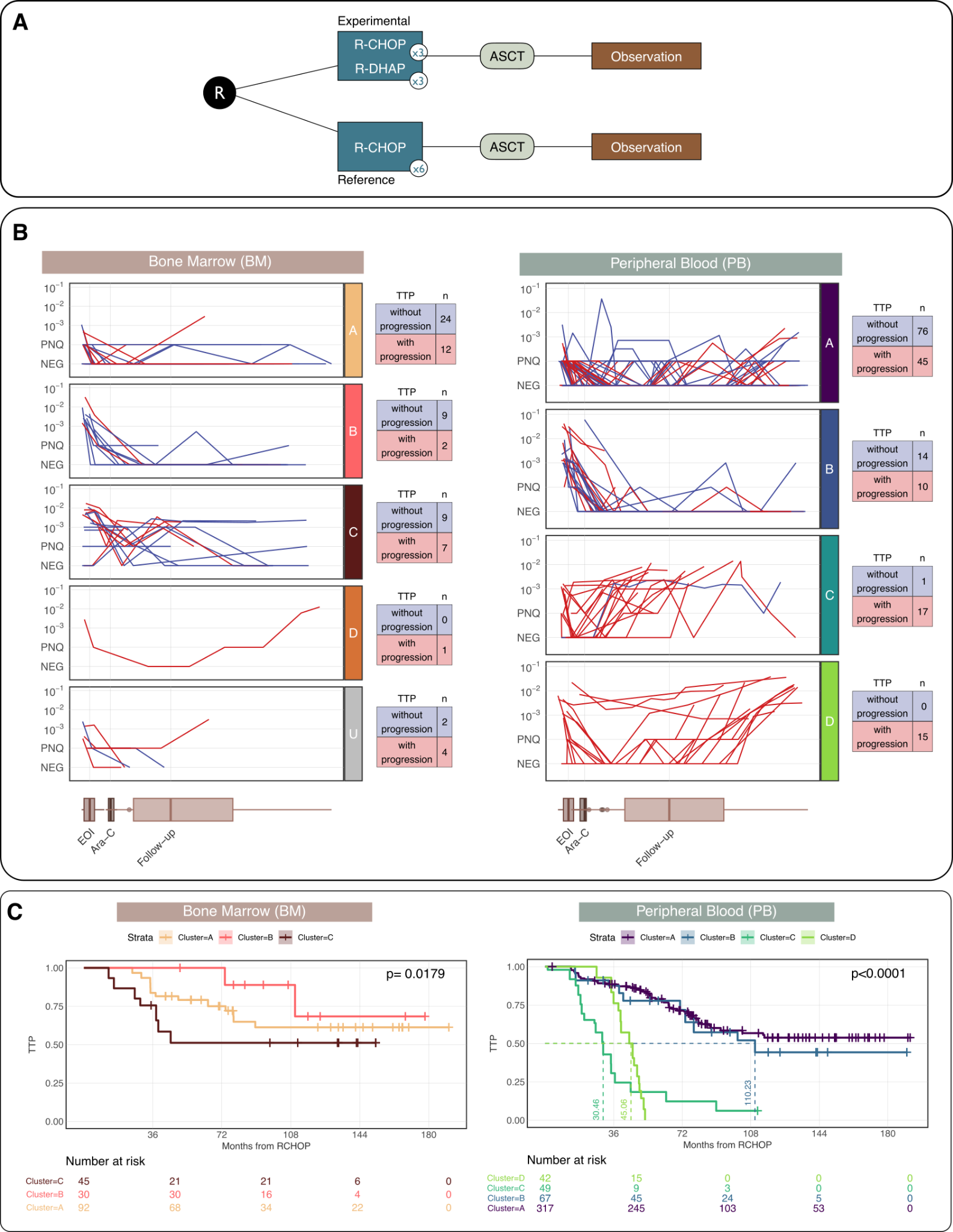
***Figure S5: Model abstraction results on MCL younger cohort. (A) The clinical trial schema (B) the MCL predictive models obtained from CONNECTOR ( C ) Kaplan Meier curves*

### **S2.4 Landmark analysis of BM MRD from MCL Younger cohort**


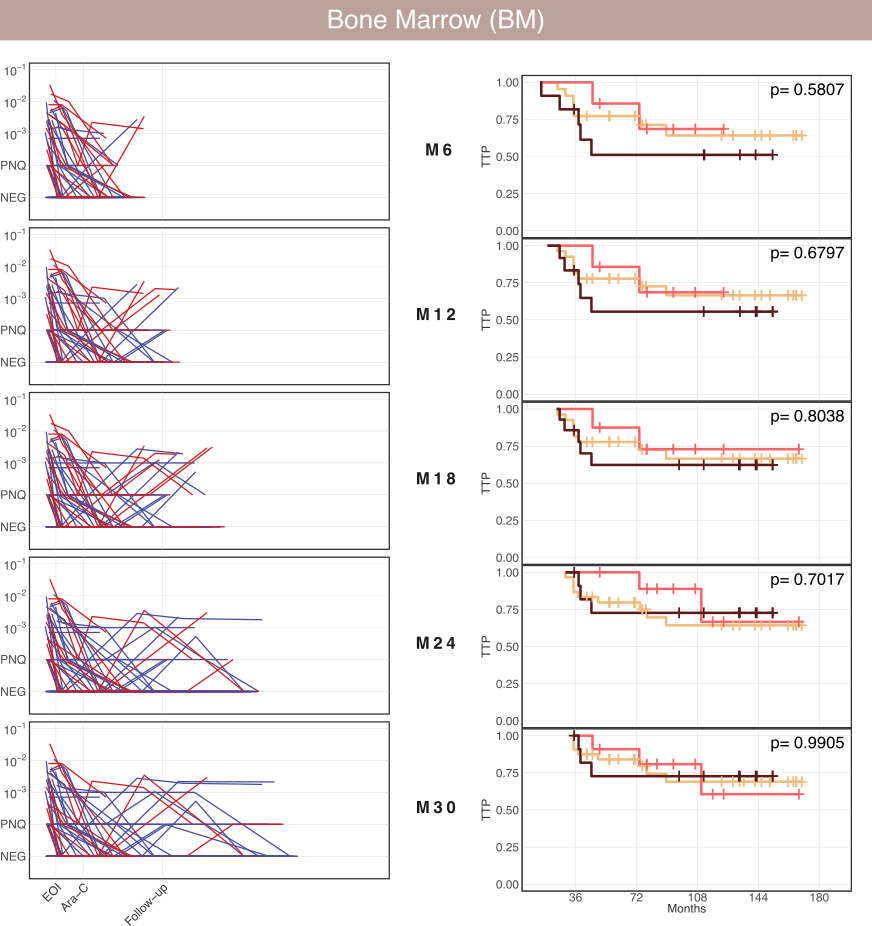


*Figure S6: Landmark analysis from the MCL Younger trial. The first column presents BM MRD data, truncated at the specified landmark points (M6, M12, M18, M24, and M30). Corresponding Kaplan-Meier curves illustrate TTP distribution, with patients stratified according to MCL predictive model classifications at each landmark point. Log-rank test results are provided in all Kaplan-Meier plots.*

### **S2.5 Overall assessment of therapy effects over MCL predictive models**

*
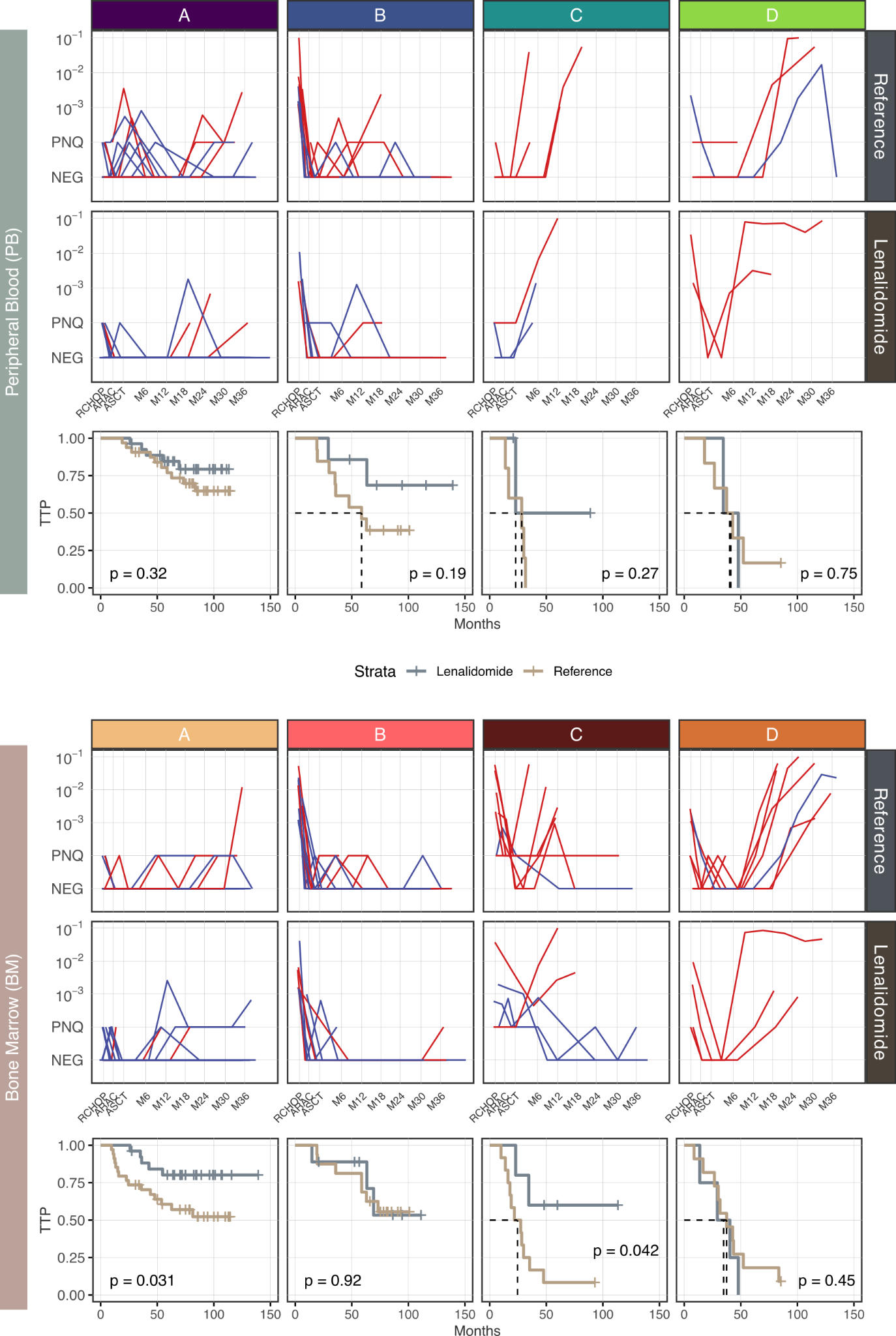
*

*Figure S7. The MCL predictive models are stratified based on whether patients received lenalidomide treatment. Kaplan–Meier survival curves are presented for each predictive model, comparing the two treatment arms. Results are shown separately for peripheral blood (PB, top panel) and bone marrow (BM, bottom panel) data.*

### **S2.8 Analysis of MRD embedding limited longitudinal sampling**

In this additional analysis, BM and PB were analyzed independently, encompassing 65 patients and yielding 17 MRD profiles in BM and 48 in PB. For BM, MRD curves with two time points include only measurements collected before randomization. Specifically, 9 patients had MRD assessed during R-CHOP and Ara-C, 6 during R-CHOP and ASCT, and 2 during Ara-C and ASCT. For PB, 48 patients with either two (n = 16) or three (n = 32) time points were included. Among patients with three time points, only one patient had all measurements collected during follow-up (M6, M12, and M18), two patients had two measurements before randomization and one during follow-up, while all remaining patients had measurements collected exclusively before randomization. All PB profiles with two time points were collected prior to randomization.

We then performed two independent CONNECTOR analyses, one for BM and one for PB.

In BM, the optimal number of clusters was three, fully recapitulating the patient stratification observed in the main manuscript (Figure S8-A). Cluster BM_1 includes patients characterized by rapid and stable MRD negativization, with early MRD values either non-quantifiable positive or showing low-level persistence. The majority of these patients did not experience relapse (51 out of 85, 60%) . Cluster BM_2 comprises patients with high MRD levels during initial treatment phases (R-CHOP/Ara-C), followed by MRD negativization at ASCT, with a balanced distribution of patients with (n=23, 54%) and without progression (n=19, 45%). Cluster BM_3 includes patients showing high initial MRD levels, transient MRD negativization, and subsequent MRD reappearance; in this group, most patients experienced relapse (n= 22 out of 28, 80%). Kaplan–Meier analysis showed that the TTP has not yet been reached for BM_1, whereas it is approximately 62 months for BM_2 and 30 months for BM_3 (Figure S8-B). Survival curves differed significantly across clusters (log-rank p < 0.0001).

Similarly, the CONNECTOR model, when independently estimated on PB samples, reproduced the stratification reported in the main paper. PB_1 includes patients with stable MRD negativization or alternating MRD trends and is enriched for patients without progression (n=70 of 121, 58%). PB_2 and PB_3 comprise patients characterized by early and late MRD reappearance, respectively, with the majority experiencing relapse(n=144 out of 19 and n=11 out of 14 in PB_2 and PB_3, respectively. Kaplan–Meier curves stratified by PB clusters (Figure xxxB) show that the median TTP has not yet been reached for PB_1, while it is approximately 49 months for PB_2 and 26 months for PB_3, with a statistically significant difference among clusters (log-rank p < 0.0001).


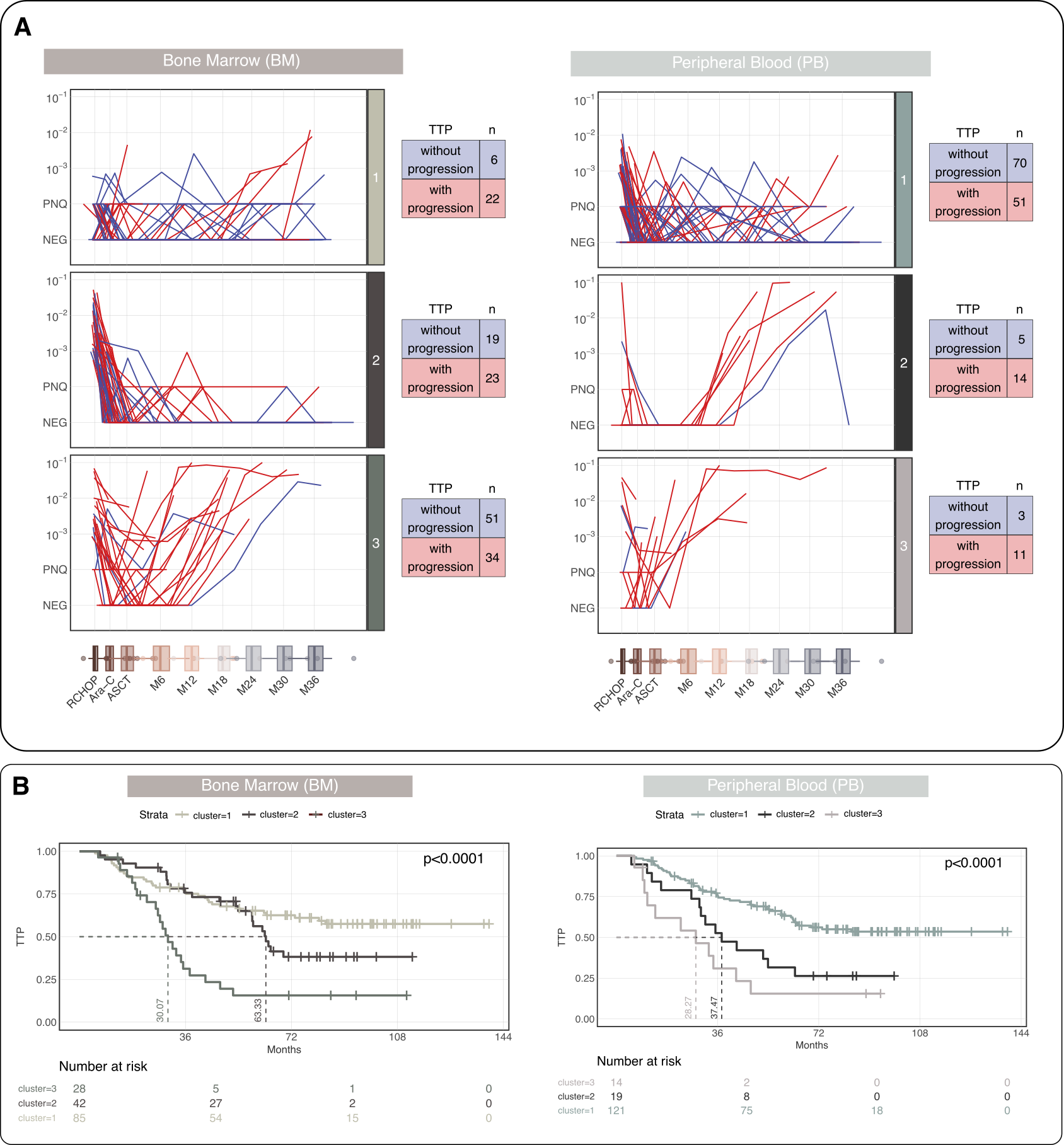


*Figure S8: Definition of MCL predictive models. (A) CONNECTOR-based analysis of BM and PB MRD data, identifying three distinct clusters. (B) Kaplan–Meier survival curves (time from R-CHOP) stratified by CONNECTOR clusters in MCL patients. Dashed lines and associated labels denote the median survival for each group, defined as the time point at which the estimated survival probability drops below 0.5.*

To further evaluate uncertainty in MRD dynamics, we include curves with two time points in BM and two or three time points in PB in the scatter plots in Figure S9-A, showing the entropy values associated with each MRD curve across the number of time points. Notably, MRD trajectories with only two time points exhibit markedly higher entropy than longer trajectories, reflecting greater uncertainty in pattern assignment. The only exception is BM_1, where entropy values remain consistently low (<0.5), which can be explained by both measurements being MRD-negative, leading to a highly stable, unambiguous trajectory. In Figure S9-B the BM and PB discriminant functions are reported. The plots suggest that the interval from M18 to M30 is crucial for defining the MCL predictive model in BM tissue (left column), while the PB discriminant functions (right column) show concordance on the importance of the M6 time point and further strengthen the relevance of the M18-M30 interval.


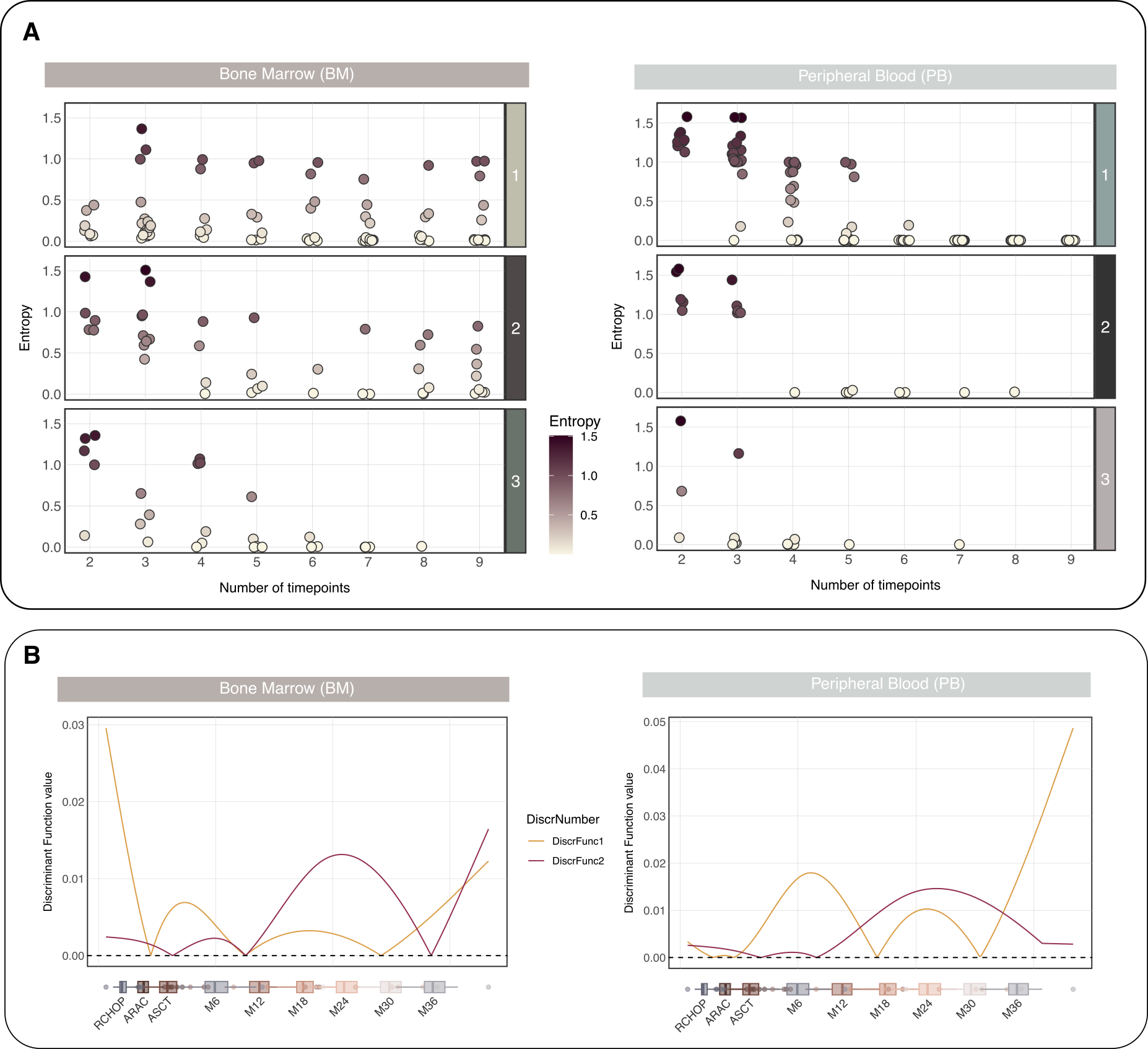


*Figure S9:* (A) *In both panels, the scatter plots display the entropy values of individual MRD trajectories (y-axis) as a function of the number of longitudinal time points available (x-axis). Higher entropy values indicate greater uncertainty in assigning MRD curves to clusters.* (B) *The line plots depict the discriminant functions across time points, where higher function values denote a stronger contribution of the corresponding intervals to the cluster assignment.*

As in the main paper, we classified longitudinal MRD measurements from the MCL Younger trial using the previously estimated predictive model derived from the FIL-MCL0208 dataset during the predictive model definition phase, considering only MRD trajectories with at least two time points. For BM, MRD data with at least two time points were available for 110 patients. Among these, 85 patients were classified as having favorable MRD kinetics (66 in BM_1 and 19 in BM_2), while 14 patients were classified as having unfavorable MRD kinetics (BM_3). Eleven patients could not be classified. Kaplan–Meier analysis showed that the median time to progression (TTP) exceeds the study duration for BM_1 and BM_2, whereas it is equal to 3.45 years for BM_3 (Figure S10-A). The estimated survival functions for the three CONNECTOR clusters differ significantly (p <0.0001).

For PB, longitudinal MRD profiles with at least two time points were available for 217 patients. Of these, 184 patients were assigned to favorable MRD kinetic patterns (PB_1), whereas 31 patients were classified as having unfavorable MRD kinetics (9 in PB_2 and 22 in PB_3). Two patients could not be assigned to any PB cluster. Kaplan–Meier analysis indicated that the median time to progression (TTP) is 9.54, 3.21, and 2.37 years for PB_1, PB_2 and PB_3, respectively (Figure S10-B). Survival curves corresponding to the three PB CONNECTOR clusters were significantly different (p < 0.0001).

*
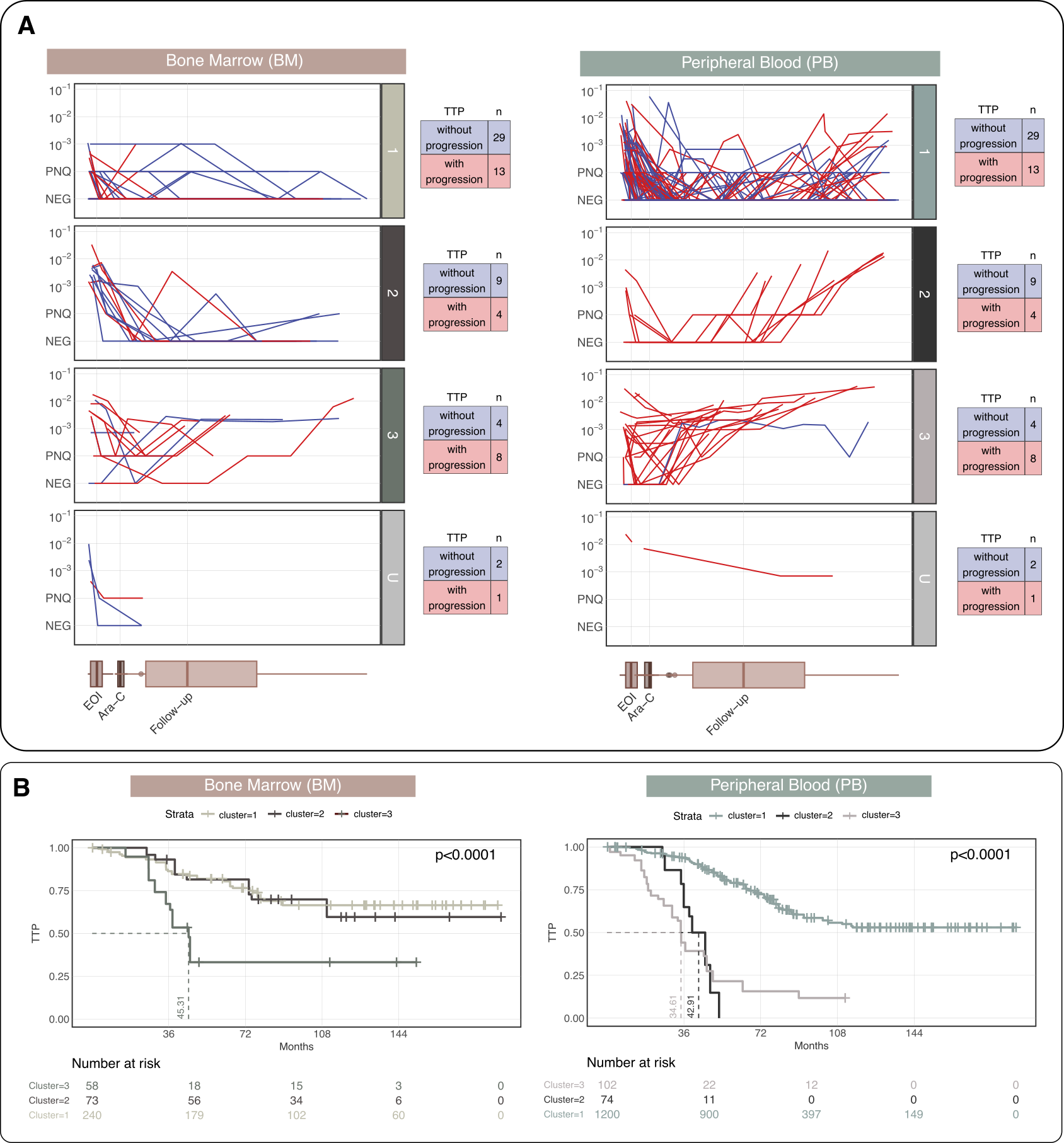
*

*Figure S10 Classification of MCL Youger trial into the MCL predictive models.*
